# Weak population spatial genetic structure and low infraspecific specificity for fungal partners in the rare mycoheterotrophic orchid *Epipogium aphyllum*

**DOI:** 10.1007/s10265-021-01364-7

**Published:** 2022-01-06

**Authors:** Julita Minasiewicz, Emilia Krawczyk, Joanna Znaniecka, Lucie Vincenot, Ekaterina Zheleznaya, Joanna Korybut-Orlowska, Tiiu Kull, Marc-André Selosse

**Affiliations:** 1 Faculty of Biology, Department of Plant Taxonomy and Nature Conservation, University of Gdańsk, ul. Wita Stwosza 59, 80-308 Gdańsk, Poland; 2 Intercollegiate Faculty of Biotechnology of University of Gdansk and Medical University of Gdansk, Abrahama 58, 80-307 Gdansk, Poland; 3 Normandie University, UNIROUEN, INRAE, ECODIV, 76000 Rouen, France; 4 Peoples’ Friendship University of Russia, Podolskoye shosse 8/5, 115093 Moscow, Russia; 5 Timiryazev State Biological Museum, Malaya Gruzinskaya, 15, 123242 Moscow, Russia; 6 Estonian University of Life Sciences, Tartu, Estonia; 7 Institut de Systématique, Evolution, Biodiversité (ISYEB), Muséum National d’Histoire Naturelle, CNRS, Sorbonne Université, EPHE, 57 rue Cuvier, CP 39 75005 Paris, France

**Keywords:** Clonality, *Epipogium aphyllum*, Gene flow, Genetic structure, *Hebeloma*, *Inocybe*, Mycoheterotrophic orchid, Mycorrhizal specificity

## Abstract

Some plants abandoned photosynthesis and developed full dependency on fungi for nutrition. Most of the so-called mycoheterotrophic plants exhibit high specificity towards their fungal partners. We tested whether natural rarity of mycoheterotrophic plants and usual small and fluctuating population size make their populations more prone to genetic differentiation caused by restricted gene flow and/or genetic drift. We also tested whether these genetic characteristics might in turn shape divergent fungal preferences. We studied the mycoheterotrophic orchid *Epipogium aphyllum*, addressing the joint issues of genetic structure of its populations over Europe and possible consequences for mycorrhizal specificity within the associated fungal taxa. Out of 27 sampled *E*. *aphyllum* populations, nine were included for genetic diversity assessment using nine nuclear microsatellites and plastid DNA. Population genetic structure was inferred based on the total number of populations. Individuals from 17 locations were included into analysis of genetic identity of mycorrhizal fungi of *E. aphyllum* based on barcoding by nuclear ribosomal DNA. *Epipogium aphyllum* populations revealed high genetic diversity (uHe = 0.562) and low genetic differentiation over vast distances (*F*_ST_ = 0.106 for nuclear microsatellites and *F*_ST_ = 0.156 for plastid DNA). Bayesian clustering analyses identified only two genetic clusters, with a high degree of admixture. *Epipogium aphyllum* genets arise from panmixia and display locally variable, but relatively high production of ramets, as shown by a low value of rarefied genotypic richness (*R*_r_ = 0.265). *Epipogium aphyllum* genotype control over partner selection was negligible as (1) we found ramets from a single genetic individual associated with up to 68% of the known *Inocybe* spp. associating with the plant species, (2) and partner identity did not show any geographic structure. The absence of mosaicism in the mycorrhizal specificity over Europe may be linked to preferential allogamous habit of *E. aphyllum* and significant gene flow, which tend to promote host generalism.

**Supplementary Information:**

The online version contains supplementary material available at 10.1007/s10265-021-01364-7.

## Introduction

Mycoheterotrophy, the ability to obtain organic carbon from mycorrhizal fungi, supports the nutrition of some achlorophyllous plants (Leake 1994). Most mycoheterotrophic plants (MHP) associate with mycorrhizal fungi, which are themselves associated with surrounding autotrophic plants; they cheat on a mycorrhizal network built on an otherwise mutualistic symbiosis, where photosynthates from autotrophic plants are exchanged for water and soil minerals collected by fungal partners (Jacquemyn and Merckx 2019; Selosse and Rousset 2011).

Mycoheterotrophic plants arose independently more than 40 times in many unrelated plant lineages (Jacquemyn and Merckx 2019; Merckx et al. 2013; Perez-Lamarque et al. 2020), thus providing fascinating models to study the evolution of plant-fungi mycorrhizal interactions. Mycorrhizal associations of MHP are usually more specialised than revealed by their relative autotrophic species (Gomes et al. 2017; Zhao et al. 2021, but see Martos et al. 2009, and Roy et al. 2009a for the tropics), with specificity levels varying between single fungal families, genera, or species, in more extreme cases (Barrett et al. 2010; Bidartondo and Bruns 2005; Selosse et al. 2002; Taylor and Bruns 1997, 1999). Two non-exclusive evolutionary processes leading to high specificity of MHP towards mycorrhizal fungi have been proposed *i.e*. more stringent MHP selection for beneficial fungi (Perez-Lamarque et al. 2020) and fungal avoidance of parasitic-like interactions with MHP (Zhao et al. 2021). Specialisation, as an adaptive process, requires genetic variation within populations, which provides trait variation upon which selection may act (Futuyma and Moreno 1988; Poisot et al. 2011). Mycoheterotrophic plants common characteristics, such as usual small population size and often fluctuating numbers of individuals (Merckx 2013) as well as frequently observed autogamy (Waterman et al. 2013) may entail lower gene flow and genetic drift. This could lead to differentiation of populations and evolution of divergent specialisation towards mycorrhizal fungi also through narrowing specialisation for fungal partner, as with the loss of genetic diversity plants may lose the ability to cheat on certain fungi (see Kennedy et al. 2011).

In orchids, which contain the largest number of MH species (> 200 species; Freudenstein and Barrett 2010; Merckx et al. 2013), genetic drift acting strongly in usually small and disjunct populations is suggested as an important speciation mechanism (the ‘drift-selection’ model; Gentry and Dodson 1987; Tremblay et al. 2005). However, population genetic studies do not support this as orchids, among herbaceous plants, recurrently show the lowest spatial genetic structure, which may be explained by efficient gene flow among populations (for review, see Phillips et al. 2012). Nevertheless, in mycoheterotrohic orchids, cryptic specialisation for fungi may arise despite high gene flow if plants’ fungal preferences are selected in a local environment throughout the species range, as expected in the geographic, mosaic model of coevolution (Thompson 2005). Unfortunately, population genetic studies that could help to elucidate the extent of genetic variation as well as patterns of gene flow for mycoheterotrophic plants (orchids in particular) are still rare (but see Alves et al. 2021; Beatty and Provan 2011a, b; Fama et al. 2021; Hopkins and Taylor 2011; Klooster and Culley 2010).

**Fig. 1:**
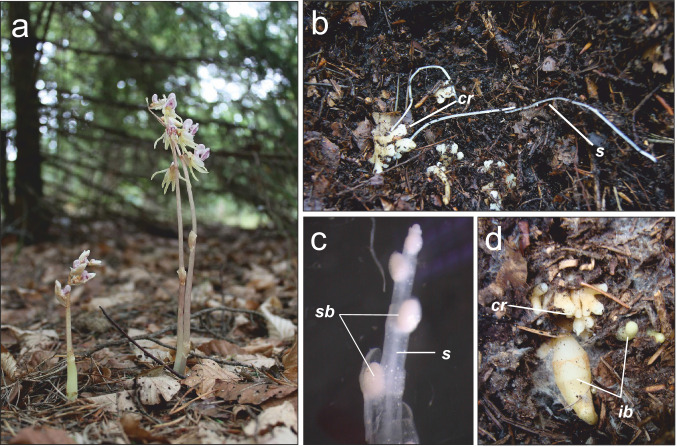
*Epipogium aphyllum* morphology*.* **a** inflorescence shoots. **b** rhizome with long running stolons. **c** apex of a stolon with bulbils, reprinted from Roy et al. (2009b; scale bar 1 mm). **d** rhizome with inflorescence buds; *cr* coralloid rhizome, *ib* inflorescence bud, *s* thin stolon, *sb* bulbil on a stolon

The rare, mycoheterotrophic orchid *Epipogium aphyllum* Sw. (Krawczyk et al. 2016) associates with many *Inocybe* species in Eurasia, and occasionally with distantly related fungi from *Hebeloma, Lactarius* and *Thelephora* (Roy et al. 2009b). The authors found no clear geographic pattern of association, though, which might result from insufficient sampling both throughout the plant range and within populations to test for ‘mycorrhizal races’ formation (see Taylor et al. 2004). The last may indeed be facilitated by clonal growth (Roy et al. 2009b; Fig. 1b–d) since selection may act on clones with higher performance in a local environment resulted from fine-tuning with locally available fungi. Alternatively, Roy et al. (2009b) finding may suggest a homogenizing effect of gene flow, which remains to be tested but allogamous habit of *E. aphyllum* along with demographic stability provided by clonal growth may indeed enhance gene flow and slow down loss of genetic diversity through genetic drift (see Barrett 2015; De Vitte and Stöcklim 2010; Honnay and Bossuyt 2005) promoting generalism in fungal association. An approach combining large-scale population genetics of MH plants along with their fungal preferences should shed more light on the evolution of specialisation in these plants.

Our study addresses the joint issues of genetic diversity, spatial genetic structure and inferred gene flow in allogamous *E. aphyllum* over Eurasia with a focus on Europe, and their possible consequences for interaction with its mycorrhizal partners. We studied intra- and interpopulation genetic diversity, using both nuclear and plastid genetic markers for complementary information (Petit et al. 2005), and we identified fungi by molecular barcoding, combining our data and samples with those of Roy et al. (2009b) and Liebel and Gebauer (2011). We addressed the following questions: (1) What is the level of genetic diversity and the extent of clonal growth of *E. aphyllum*? (2) Does genetic structure imply isolation of sub-populations, and potential cryptic speciation in *E. aphyllum*? (3) Does the mycorrhizal interaction display finer specificity for *Inocybe* subgenera or rare mycorrhizal partners in local populations?

## Materials and methods

### Study species

*Epipogium aphyllum* (Fig. 1a) is a rare, boreal to montane MH orchid found throughout Eurasia, but more abundant in Europe (Hultén and Fries 1986; Vakhrameeva et al. 2008). Despite a wide Eurasian distribution, it belongs to the most threatened European orchids (Kull et al. 2016) and is protected or placed on the IUCN red list in most of the 56 countries (Govaerts et al. 2018). Over its entire range, *E. aphyllum* grows from lowlands to high mountains (Taylor and Roberts 2011). It is found in beech, oak, fir, and spruce forests on neutral to alkaline soils (Binkiewicz 2014; Hereźniak and Piękoś-Mirkowa 2014). Its populations usually comprise a few to a dozen shoots, rarely more (> 100 shoots; Irmisch 1853; Kuszaj et al. 2011; Święczkowska 2010; Taylor and Roberts 2011). Throughout its life cycle, the plant depends on ectomycorrhizal *Inocybe* fungal species*,* although rare associations with *Hebeloma*, *Lactarius* and *Thelephora* have also been reported (Jąkalski et al. 2021; Liebel and Gebauer 2011; Roy et al. 2009b). Flowering stems (5–25 cm) appear occasionally above-ground (Taylor and Roberts 2011). The flowering is highly unpredictable, usually in July and August, yet with possible early (June) and late (November) flowerings (Święczkowska 2010). Flowers produce many chemical attractants (Jakubska-Busse et al. 2014) and nectar (Krawczyk and Kowalkowska 2015) and are most probably pollinated by bumblebees (Jakubska-Busse et al. 2014; Krawczyk 2016). Plants are self-compatible, but no spontaneous autogamy has been reported so far (Krawczyk et al. 2016). Like all orchids, *E. aphyllum* produces numerous dust-like seeds capable of long-distance, wind-borne dispersal (Arditti and Ghani 2000; Rakosy 2014).

### Sample collection

**Fig. 2:**
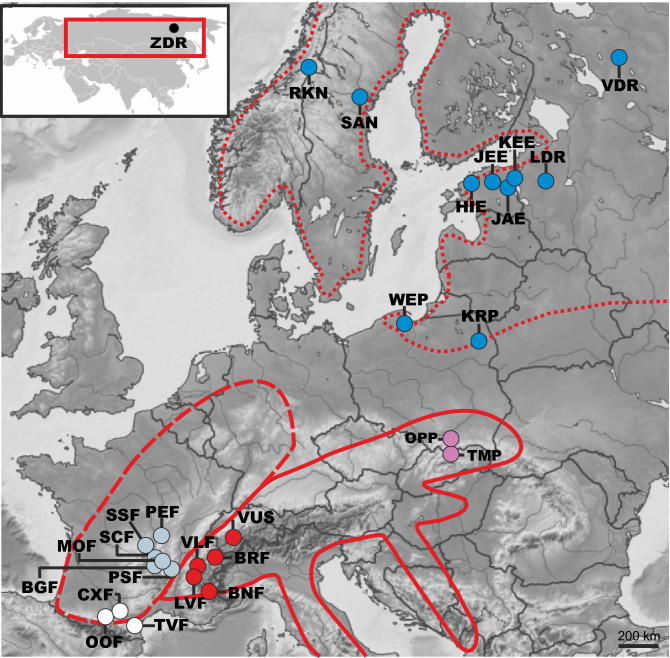
Location of *E. aphyllum* populations sampled for the study. Inset presents the Asian (Eastern) part of the species range. Three-letter codes refer to population names (see Table S1) with distinct colours for the six geographical regions (see Table S1): Pyrenees, white; Massif Central, grey; Alps, red; Carpathians, pink; Baltic Region, blue; Asia, black. The red line displays the European species disjunctive range from Taylor and Roberts (2011). Three main parts of the range are delineated in red: Western (dashed line), Southern (solid line) and Northern (dotted line)

Samples were obtained from 26 locations spanning the European part of *E. aphyllum*'s range, and one site from the eastern, Asian edge of the distribution area (Fig. 2; Table S1). Samples from 15 populations were collected between 2011 and 2017. To avoid redundant sampling of multiple ramets of the same genetic individual (genet), one flower was collected per shoot (ramet) emerging at least 1 m apart, before drying in silica gel. Furthermore, DNA isolates from Roy et al. (2009b; 10 French populations) and Liebel and Gebauer (2011; one Norwegian population) as well as one sample from Sweden (Jodrell Kew DNA bank, sample No. 19248) were added for a total of 248 samples. Due to (i) the species’ rarity and protected status, and (ii) ephemeral flowering and fluctuating shoot number, the numbers of samples per site vary between 1 and 80 (with median value equals four, see Table S1 for details on provenance and afforded sampling). Additionally, to avoid bias in genetic metrics value caused by clonality, we applied calculation based only on clone-corrected sample sets, which additionally lowered the number of samples available for analyses. To maximise analytical output for this unequal sample size while still conforming to the analytical rules, we applied analyses in two data pools which are described in detail in the following sections.

To study whether mycorrhizal interactions displayed finer specificity for fungi in a local population, we harvested a rhizome fragment from 40 out of the 80 individuals sampled for genetic diversity within a large Wejherowo (WEP) population. Root samples were also obtained in Ojcowski National Park (OPP; 4 samples), Tatra Mountains (TMP; 6 samples) and Kozi Rynek (KRP; one sample). In WEP, KRP and 2 plants from TMP one rhizome fragment (0.5 cm long) was harvested per plant (in compliance with obtained sampling authorisation) by digging soil *ca*. 15 cm along on one side of the stem and carefully reaching a side of the rhizome. After sampling, the hole was refilled (a protocol allowing plant survival modelled after Roy et al. 2009b). In four samples from OPP and TMP authorized destructive sampling allowed pooling at least 5 rhizome fragments per plant. Within 1 h after harvesting, rhizome fragments were washed with water, sterilised in 70% EtOH and dried in silica gel. Additionally, plants from Roy et al. (2009b) and Liebel and Gebauer (2011) were genotyped and included into the analyses. We did not include a sample representing *Tomentellopsis* sp. in the analyses, as it was found only once in our study and its mycorrhizal status requires further confirmation. The same attitude was presented by Roy et al. ([Bibr CR104][Bibr CR105]) in the case of *Lactarius* sp. and *Thelephora* sp.

### Microsatellite genotyping

Total genomic DNA from flowers and rhizome samples was extracted with a CTAB-based method (Bekesiova et al. 1999). In total, 248 DNA samples were analysed for neutral genetic diversity with 13 nuclear microsatellite loci (nSSRs; Table S2) developed for this study using MiSeq sequencings, applying methods outlined in Minasiewicz and Znaniecka (2014) based on an *E. aphyllum* samples from Wejherowo (WEP). Amplifications were performed in 10 μL of reaction mix containing: 2 × Type-it Mix (Qiagen, Germany), 0.4 mM of fluorescently labelled forward primer, 0.4 mM of reverse primer and 50 ng of DNA template. The following PCR conditions were used: 5 min of initial denaturation at 96 °C followed by 35 amplification cycles (95 °C for 30 s, primer-specific annealing temperature (Table S2) for 30 s, then 72 °C for 45 s), and a final extension step of 72 °C for 10 min. Genotyping was performed on an ABI 3130 sequencer (Applied Biosystems) with LIZ-500 size standard (Applied Biosystems). Alleles were manually scored, and genotypes were determined for each individual, using GeneMapper™ (Applied Biosystems).

### Plastid locus sequencing

Variable plastome size reduction and rearrangements accompanying loss of photosynthesis (Fengy et al. 2016) hinder the use of common plastid markers in MH plants (Cameron 2004). Thanks to the availability of two plastid genomes of *E. aphyllum* (GenBank accessions KJ946456 and KJ772292; Schelkunov et al. 2015), we designed primers for three variable fragments in conserved regions of housekeeping genes: *accD*-*trnE* intron (555 bp), *rps4* (478 bp) and *rps12-trnL* intron (564 bp; Table S3)*.* In total, 158 samples were sequenced for those three regions. PCR amplification was carried out in 10 μL of reaction mix containing: 1 × MyTaq HS Mix (Bioline, UK), 0.35 mM of both primers using parameters described in Table S4. Amplicons were purified with Exo-BAP (Eurx, Poland) and sequenced as in Selosse et al. (2002) on an ABI 3720 automated capillary DNA Sanger sequencer. The complementary strands were assembled by AutoAssembler (ABI). All sequences were aligned using SeaView v.4 (Gouy et al. 2010), and manually corrected, before deposition in GenBank under accession numbers MK201804 to MK202010.

### Sequencing of plant and fungal nuclear ITS

Roy et al. (2009b) showed that nearly 90% of all obtained fungal sequences and in particular all found in pelotons belonged to ectomycorrhizal Basidiomycetes, which was further confirmed by the presence of hyphae with clamp connections in pelotons. Since the aim of our study was to investigate the level of specificity of the mycorrhizal association of *E. aphyllum* we selected methods that enabled efficient screening for fungi from Basidiomycetes. We amplified the Internal Transcribed Spacer (*ITS*) region of fungal rDNA using the primers ITS1F and ITS4B designed for Basidiomycetes (Gardes and Bruns 1993). Amplification and sequencing steps were carried out following the PCR conditions of Gardes and Bruns (1993). We applied direct sequencing from roots whenever this was possible, otherwise PCR products were cloned as in Selosse et al. (2004) and at least 5 clones per plant were sequenced as above. Compared with NGS sequencing, we found this approach to be time- and cost-efficient, while also consistent with the main objective of the study. Edited sequences (or consensus from clones) were deposited in GenBank (KX867464-503 and OL461961-71).

To ensure conspecificity of *E. aphyllum* samples, the *ITS* locus was PCR-amplified from 44 plants from 18 localities (Table S1) with primers 17SE and 26SE (Sun et al. 1994) targeting DNA fragments encompassing the one sequenced by Roy et al. (2009b). Amplifications were carried out as above following PCR conditions by Sun et al. (1994), and sequences obtained as above were deposited in GenBank (MK450367-410).

### Analyses of local genetic diversity of sampled populations

#### Nuclear microsatellite markers

Prior to analyses, we checked for the occurrence of genotyping errors and null alleles using micro-checker 2.2.3 (Van Oosterhout et al. 2004). The discriminative power of the present set of loci was checked with *R* (R Core Team 2020) package poppr version 2.9.1 (Kamvar et al. 2014) to ensure its capacity to estimate the real number of multilocus genotypes (MLG) in the dataset. Assignment of samples to MLGs accounting for somatic mutation and potential scoring errors along with the number of expected MLGs at the smallest sample size ≥ 10 based on rarefaction was also carried out in poppr v. 2.9.1. To assess the probability that identical MLGs may originate from a distinct sexual reproduction event, *P*_*sex*_ was calculated in geneclone 2.0 (Arnaud-Haond and Belkhir 2007). If *P*_*sex*_ > 0.05, duplicated MLGs were treated as different individuals; otherwise, duplicated MLGs were considered as belonging to the same genet. Clonal diversity was assessed within each population by estimating genotypic richness calculated as *R* = (*G* − 1)/(*N* − 1) and defined as the estimated proportion of unique genets (*G*) in the total number of sampled ramets (*N*). In clonal organisms the value of *R*, as a function of sample numbers, follows a power law distribution, sample sizes should be as large as possible to ensure the stability of *R* values (Arnoud-Haond et al. 2007; Eckert 2002). Therefore, we also calculated less biased metrics in populations with ≥ 10 samples “*R*_r”_, where the number of genets (G) was estimated as the expected number of MLGs after rarefaction (eMLG). We averaged values of *R*_r_ and *R* (for comparison with other studies) for populations with ≥ 10 samples.

For subsequent analyses, clone-corrected data-sets were produced, where samples representing replicated MLGs were removed based on nSSR and consistently on plastid markers. Although in some instances non clone-corrected data may be applied in Hardy–Weinberg based calculations (despite violation of the model’s explicitly assumptions that precluded the use of the same genetic individual more than once; Douhnikoff and Laventhal 2016), we decided to apply a more stringent attitude to diminish possible interpretative problems connected with the influence of diverse populations’ clonality on genetic diversity parameters.

Due to the reduction of sample size by clone correction and overall small and uneven-sized populations, two, nonexclusive samples grouping were applied: “clone corrected, complete sample set” (82 MLGs for nSSRs and 69 plastid haplotypes) for analysis of genetic structure and genetic diversity at a regional level and “clone-corrected, population sample set” consisting of 9 populations with > 4 individuals each (54 MLGs for nSSRs and 50 plastid haplotypes) to study genetic structure and genetic diversity at a population level (Table S1), based on which inbreeding coefficient *F*_*I*S_ was calculated in fstat v. 2.9.3 (Goudet 2001); nuclear microsatellite diversity characterised by observed (H_o_) and unbiased expected (uH_e_) heterozygosity, as well as the number of private alleles specific to a population (pA), was calculated in GenAlEx v. 6.5 (Peakall and Smouse 2012). Allelic richness (A_R_) and private allelic richness (pA_R_) were calculated in hp-rare (Kalinowski 2005), which uses the rarefaction method to correct for sample size differences. Deviations from Hardy–Weinberg and linkage disequilibrium (LD) were calculated in arlequin v. 3.5 (Excoffier and Lischer 2010), with statistical significance assessed with 10,000 permutations, and standard Bonferroni correction was applied to obtain the appropriate significance for multiple comparisons (Rice 1989).

#### Plastid markers

Plastid haplotypes (Table S5) were assigned based on the combination of alleles at polymorphic sites (substitutions and insertion/deletion) using GenAlEx v.6.5 (Peakall and Smouse 2012). Populations from the clone-corrected, population sample set were characterised for haplotype diversity, the number of haplotypes and haplotype richness after rarefaction with CONTRIB program (Petit et al. 1998). The pattern of evolutionary branching of haplotypes and their geographical distribution were explored on a median-joining phylogenetic network tree of haplotypes (Bandelt et al. 1999) built with NETWORK 5.0.0.4. (www.fluxus-engineering.com). To avoid possible homoplasy, microsatellite regions (A_(n)_ and T_(n)_ patterns) in the *rps12*-*trnL* intron were excluded. The shortest possible tree was calculated by using the minimum spanning network pre-processing and the maximum parsimony heuristic search post-processing.

### Spatial genetic structure amongst sampled populations

Nuclear and chloroplast DNA differentiation between populations and regions was estimated using pairwise *F*_ST_ indices and analysis of molecular variance (AMOVA) to partition genetic variation within and among populations in two sets: (1) the clone-corrected, population sample set and (2) the clone-corrected, complete sample set pooled into 6 regional groups according to their geographic provenance (Pyrenees, Massif Central, Alps, Carpathians, Baltic Region and Asia; Table S1, Fig. 1). Those geographical regions play a key role in glacial and postglacial history of European flora, enabling interpretation of the data in a well-documented frame (Hewitt 2000; Taberlet et al. 1998). Samples were pooled to comply with minimal sample size to run AMOVA analyses and to study the pattern of gene flow based on all collected (but clone-corrected) samples despite their sometimes small number per population. These calculations were performed in arlequin 3.5 (Excoffier and Lischer 2010) with 10,000 permutations to support statistical significance. Unfortunately, overall small sample size per population and high clonality did not allow further assessment of gene flow applying Bayesian inference.

Isolation by distance (IBD) among 9 populations was tested for plastid and nuclear markers with a Mantel test (Mantel 1967) on the clone-corrected, complete sample set in GenAlEx. We compared matrices of genetic *versus* geographic distance between populations (Slatkin 1993).

To characterise spatial genetic structure amongst populations over Eurasia, the Bayesian clustering method implemented in structure 2.3.3 (Pritchard et al. 2010) was applied to the clone-corrected, complete sample set. Ten separate runs of structure were performed for possible K values ranging from 1 to 10, with a burn-in of 500,000 iterations and run-length of 1,000,000 Markov chain Monte Carlo iterations per each run. The admixture model assuming correlated allele frequencies was applied, with location prior. The optimal K was determined by the Evanno method (Evanno et al. 2005) using structure harvester (Earl and von Holdt 2012). clumpp software (Jakobsson and Rosenberg 2007). Jakobsson and Rosenberg (2007) was used to average results for each K value across runs, and summary bar plots were built using distruct v.1.1 (Rosenberg 2004). To further understand the clustering patterns, genetic distance-based principal coordinate analysis (PCoA) was carried out using GenAlEx v. 6.5, also for the clone-corrected, complete sample set. This multivariate descriptive method is free of any model assumption and can thus usefully validate the Bayesian clustering output (Patterson et al. 2006).

### Fungal identification and phylogenetic analyses

To identify fungi associated with *E. aphyllum* rhizomes, ITS sequences were compared by BLAST to the UNITE database (Kõljalg et al. 2013). Since the high variability of ITS in *Inocybe* makes alignment difficult in the *Inocybe* genus, we estimated phylogenetic relationships in two ways. Sequences were clustered into operational taxonomic units (OTUs) at a 3% sequence divergence threshold used as a proxy for species in *Inocybe* (Matheny 2005).

First, we applied the phylogenetic approach of ‘alignment groups’ (AGs), formed on joint 5.8S and LSU alignment of the fully identified *Inocybe* sequences established by Ryberg et al. (2008): based on the closest BLAST match preferentially obtained from identified fruitbodies, we looked for conspecific sequences across 16 AGs. Sequences from the present study were then aligned with their closest-matching sequences in the corresponding AG sensu Ryberg et al. (2008) using SeaView v. 4 (Gouy et al. 2010). The alignment for each group was corrected manually and then analysed as in Ryberg et al. (2008) under the parsimony criterion in PAUP* using 1,000 random additional repeats and TBR branch swapping. We also included in the procedure *Inocybe* sequences obtained from *E. aphyllum* by Roy et al. (2009b) and by Liebel and Gebauer (2011).

Second, we aligned selected sequences from Roy et al. (2009b), Liebel and Gebauer (2011) and sequences obtained in the present study with their closest BLAST matches obtained from identified fruitbodies, to enable a global comparison and test for geographic distribution of the symbionts. A dendrogram was built with neighbour-joining analysis (Saitou and Nei 1987) in PAUP 4.0. Genetic distances were estimated by Maximum Likelihood using a K81uf + I + G DNA substitution model chosen using a series likelihood-ratio test in Modeltest 3.7 (Posada and Crandall 1998). Midpoint rooting was applied and support was assessed with 1,000 bootstrap replicates. Spatial divergence of OTUs was tested with a Mantel test (Mantel 1967) by comparison of matrices of pairwise phylogenetic distances between fungal OTUs *versus* their hosts’ geographic distance. These calculations were carried out separately on the (1) total datasets and (2) including only taxa belonging to *Inocybe*.

## Results

### Nuclear genetic diversity

*E. aphyllum* ITS sequences differed among populations by less than 0.4% (Fig. S1), and no excess of failed amplification occurred for nSRR loci in any population (Online resources 3), supporting conspecificity of *E. aphyllum* Eurasian samples. Multilocus genotypes of 248 samples from 27 *E. aphyllum* populations were successfully amplified for 9 nSSRs (Table S1), yielding 60 alleles in total. We discarded four other loci due to missing data, or evidence for null alleles in all populations (Table S2). Permutation test in R package Poppr showed that the combination of the nine remaining loci was powerful enough to discriminate all distinct MLGs (Fig. S2). Within all sampling sites, all individual shoots with similar MLGs were unlikely to result from sexual recombination (P_sex_ < 0.05) and were thus considered as ramets of the same genet, which was further supported by the sharing of identical plastid haplotypes (see Online resources 3). Clonal diversity varied between populations, ranging from *R*_r_ = 0 in WEP (where all 80 samples represented the same genetic individual with heterozygosity at 6 out of 9 loci, so that the population is not monomorphic and we truly face a single clone; see Online resources 3), to *R*_r_ = 0.778 in ZDR; Table S1, Online resources 3) with an average of *R*_r_ = 0.265 (respectively *R* = 0.328). Repeated sampling of a ramet from the same genet was omitted from subsequent analyses performed on clone-corrected data, *i.e.* 82 distinct MLGs (Table S1), none of which were shared between populations.

All nine populations of *E. aphyllum* from the clone-corrected, population sample set (*i.e.* populations with ≥ 4 MLGs) exhibited similar, moderate to high levels of nuclear genetic diversity (Table 1). All loci were polymorphic with mean observed heterozygosity H_o_ = 0.460 ± 0.024, and unbiased expected heterozygosity uH_e_ = 0.562 ± 0.020. The populations had rather low levels of allelic richness after rarefaction, ranging from A_R_ = 2.22 in TVF to A_R_ = 3.14 in ZDR. Calculations based on population level (clone-corrected, population sample set) and regional level (clone-corrected, complete sample set) pooling populations into six regions; see their delineation in Fig. 2 and Table S1) revealed similar levels of genetic diversity (Tables 1 and 2), albeit with a tendency to decrease in Western (TVF, MOF) and Northern (LDR, JEE) populations in comparison with Southern (VUS, OPP) and Eastern ones (ZDR). The number of unique alleles after rarefaction differed among populations, with the lowest for Pyrenees, Massif Central and Baltic Region populations, and the highest values in Alpine, Carpathian and Eastern populations (Tables 1 and 2).

**Table 1:** Genetic diversity of 9 studied *E. aphylllum* populations ≥ 4 multilocus genotypes (MLG) forming clone-corrected, population data set

Region	Population	N	N _MLG_	A_R_	pA_R_	H_o_	uH_e_	*F*_IS_	N_H_	H	PH	H_R_	HD
Pyrenees	TVF	4	4	2.22	0.06	0.333	0.437	0.055	3	1	0	0.000	0.000
Massif Central	MOF	28	5	2.39	0.00	0.467	0.533	0.020	20	3	0	1.400	0.800
	BNF	4	4	2.78	0.15	0.444	0.611	0.134	4	4	2	2.000	1.000
Alps	VUS	6	6	2.61	0.17	0.448	0.512	0.008	6	5	3	1.800	0.933
Carpa thians	OPP	28	11	2.95	0.31	0.509	0.605	0.093	25	5	1	1.442	0.800
	TMP	29	9	2.73	0.13	0.514	0.534	-0.002	26	5	1	1.589	0.857
Baltic region	JEE	7	5	2.62	0.01	0.422	0.580	0.187	2	1	0	0.000	0.000
	LDR	4	4	2.78	0.06	0.417	0.611	0.221	4	1	0	0.000	0.000
Asia	ZDR	10	8	3.14	0.27	0.583	0.633	0.014	7	7	6	2.000	1.000

Parameters calculated for nSSR. *N* number of samples, *N*_MLG_ number of MLG per population, *A*_*R*_ allelic richness after rarefaction, *pA*_*R*_ private allele (alleles specific to population) richness after rarefaction,  *H*_o_ observed heterozygosity, *uH*_*e*_ unbiased expected heterozygosity, and *F*_IS_ inbreeding coefficient

Parameters calculated for plastid DNA: *N*_*H*_ number of samples, *H* number of different haplotypes, *PH* private haplotypes, *H*_*R*_ haplotype richness, *HD* haplotype diversity

**Table 2:** Genetic diversity of studied *E. aphylllum* populations (clone-corrected complete data set) grouped in 6 geographic regions

Regions	N_MLG_	A_R_	pA_R_	H_o_	uH_e_	N_H_	H	PH	H_R_	HD
Pyrenees	6	2.89	0.07	0.407	0.527	5	1	0	0.000	0.000
Massif Central	15	3.08	0.22	0.474	0.563	11	5	1	2.229	0.764
Alps	14	3.09	0.31	0.444	0.569	12	9	5	3.263	0.909
Carpathians	20	3.33	0.35	0.503	0.597	19	7	3	2.669	0.842
Baltic region	19	3.45	0.16	0.424	0.624	15	4	1	1.606	0.657
Asia	8	3.54	0.51	0.583	0.633	7	7	6	4.000	1.000

Parameters calculated for nSSR: *N* number of samples, *MLG* number of MLG per population, *A*_*R*_ allelic richness, *pA*_*R*_ private allele (alleles specific to population) richness, *H*_*o*_ observed heterozygosity, *uH*_*e*_ unbiased expected heterozygosity

Parameters calculated for plastid DNA: *N*_*H*_ number of samples, *H* number of different haplotypes, *PH* private haplotypes, *H*_*R*_ haplotype richness, *HD* haplotype diversity

For all local populations, inbreeding was low, with an average *F*_IS_ = 0.094 ± 0.048, which is not significantly different from 0 (Table 1). Accordingly, no population deviated from Hardy–Weinberg equilibrium, except LDR (Chi^2^ = 33.74, *p* = 0.014). There was no linkage disequilibrium between loci after Bonferroni correction, neither within the clone-corrected, population sample set, nor after pooling all samples (except for the loci Epi-8 and Epi-17 in OPP only).

### Plastid genetic diversity

**Fig. 3:**
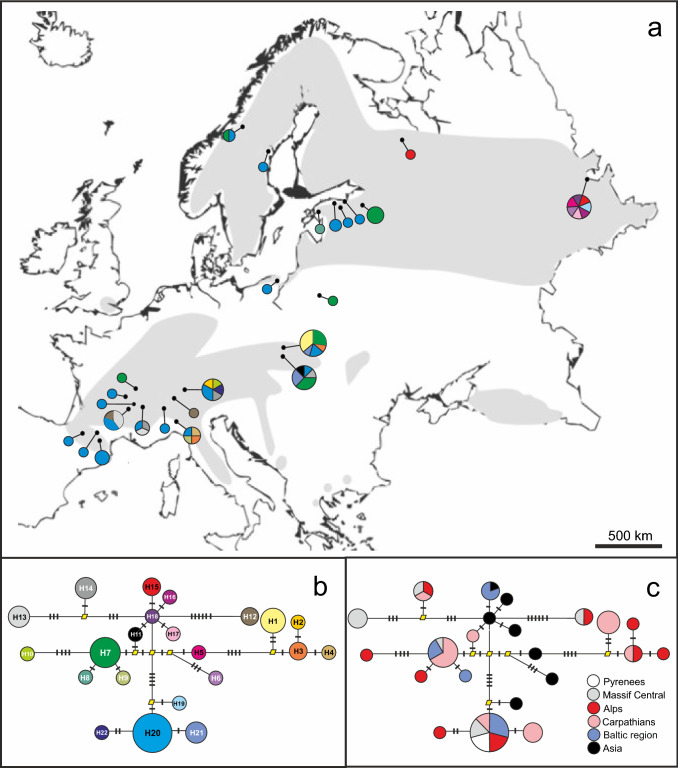
The 22 plastid haplotypes from *E. aphyllum* populations over Eurasia. **a** Frequency and distribution of haplotypes, with frequencies provided as a pie chart per location (for population name see Fig. 2), their colours corresponding with respective haplotype type from the panel b. Circle size reflects the number of unique haplotypes per population (one for the smallest circle to 11 for the biggest). The grey area indicates species disjunctive range from Taylor and Roberts (2011). The map is distorted to include the easternmost population. **b** Phylogenetic relationship between the 22 haplotypes H1 to H22. Circle size indicates frequency of haplotypes in clone-corrected data. The yellow diamonds indicate undetected, intermediate haplotype states. Cross hatches along the lines connecting the haplotypes indicate the number of mutations required for transitions between haplotypes. **c** Distribution of haplotypes among the six geographical groups of populations (see Table S1)

The 1593 bp concatenation of the three amplified plastid loci, all of which were polymorphic, revealed 22 plastid haplotypes among 158 accessions from 25 populations (Fig. 3b), twelve of which were represented by a single individual. Individuals from same nuclear MLG always revealed the same plastid haplotype, confirming the consistency of MLG delimitation (Online resources 3). Population differentiation for plastid markers followed a pattern similar to that of nuclear ones. Populations had 1 to 7 haplotypes (Fig. 3a); haplotype richness varied from H_R_ = 0 in WEP (Table 1), where 32 samples all yielded a single haplotype, to H_R_ = 2 in ZDR, where each of the 7 tested samples differed by plastid haplotype. Haplotype diversity (HD) was also variable, ranging from 0 to 1, but in the clone-corrected, population sample set, 6 out of 9 populations displayed more than one haplotype and a higher HD (0.90 on average). The most diverse populations were from the Alps (VUS, BNF), Carpathians (TMP, OPP) and Asia (ZDR), while the less diverse were Pyrenean (TUV) and from the Baltic region (Tables 1 and 2).

### Geographical structure

In the haplotype network based on plastid markers (Fig. 3b), core network positions were in most cases represented by non-observed, inferred ancestral haplotypes. However, haplotypes from the eastern part of the species range (ZDR) occupied more central positions in the network (Fig. 3c), while haplotypes immediately surrounding the core were present throughout the European range. The haplotypes at most distal positions in the network, likely the youngest, were present in all regions but the Pyrenees, with the highest abundance in the Alps. Among all populations from the clone-corrected, population sample set, there was a moderate and statistically significant plastid differentiation (*F*_ST_ = 0.156, *p* < 0.001; Table 3), but this pattern was mostly driven by high pairwise *F*_ST_ values involving LDR (Northern) and ZDR (Eastern; Table S6). When pooling samples from the clone-corrected, complete sample set into 6 regions (Table 3), differentiation levels were lower than between the populations of the clone-corrected, population sample set, although still significant (*F*_ST_ = 0.112, *p* < 0.001). Pairwise comparisons consistently revealed a significant differentiation of Pyrenean populations (Western) from Eastern (*F*_ST_ = 0.434) as well as Southern populations (Alps, *F*_ST_ = 0.189, and Carpathians, *F*_ST_ = 0.275), while other comparisons revealed low or non-significant differentiation (Table S7).

**Table 3:** Analysis of molecular variance (AMOVA) of *E. aphyllum* for nuclear microsatellites and plastid DNA for nine populations ≥ 4 MLG and pooled data set into six geographic regions (Pyrenees, Massif Central, Alps, Carpathians, Baltic Region, Asia)

Molecular marker	Source of variation	df	*F* statistics
**Microsatellite DNA**			
9 populations ≥ 4 MLG	Among populations	8	0.106***
	Within populations	101	0.894
6 geographical regions	Among regions	5	0.047***
	Within regions	158	0.953
**Plastid DNA**			
9 populations ≥ 4 MLG	Among populations	8	0.156***
	Within populations	41	0.844
6 geographical regions	Among regions	5	0.112***
	Within regions	63	0.829

***Statistical significance *P* < 0.001

Nuclear markers showed similar patterns, with moderate level of genetic differentiation when calculated from the clone-corrected, population sample set (Table 3; global *F*_ST_ = 0.106), and very low, although significant, when calculated from the clone-corrected, complete sample set pooled into six regions (Table 3; *F*_ST_ = 0.044). Pairwise comparisons between populations or regions (Tables S6 and S7) showed the highest and significant differentiation between the populations from the Western edge of the range, *i.e.* Pyrenees and Massif Central (*e.g.* TVF, MOF) and the Eastern one (ZDR).

**Fig. 4:**
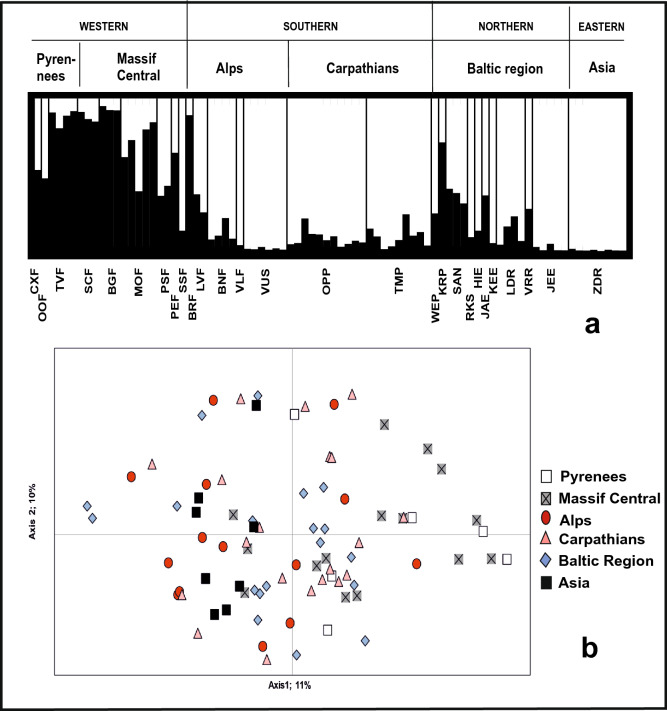
Clustering analyses of *E. aphyllum* based on the 82 MLGs data, and considering the six geographical groups of populations (see Table S1). **a** The two clusters found with the software structure. Bars indicate individual probability of membership in black (cluster #1) and white (cluster #2). **b** PCoA of nSSR genetic diversity, with regional origin of MLGs

Structure analysis for the clone-corrected, complete sample set (82 MLGs) showed the best clustering for K = 2 clusters (Fig. 4a, Figs. S3, S4) with (i) admixture in all populations and (ii) a geographical pattern distinguishing Pyrenees and Massif Central (West Europe) from the other populations. Analysis by PCoA further confirmed this trend, but with high overlapping of individuals from various parts of the range (Fig. 4b): while explaining 21% of variation in nSSR data only, PCoA also highlighted a limited spatial genetic structure, as the first axis separated westernmost populations (Pyrenees and Massif Central) from the easternmost ones (ZDF; Fig. 4b). No significant IBD was found neither based on SSRs (Fig. S5) nor plastid DNA data (not shown).

### Diversity of mycorrhizal fungi

**Fig. 5:**
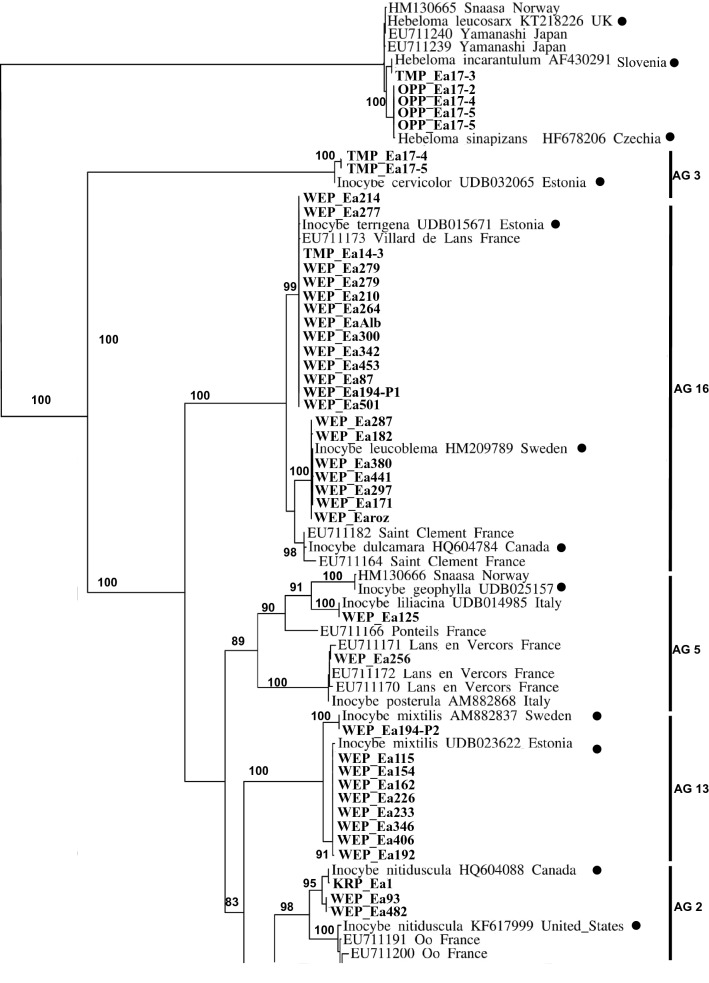

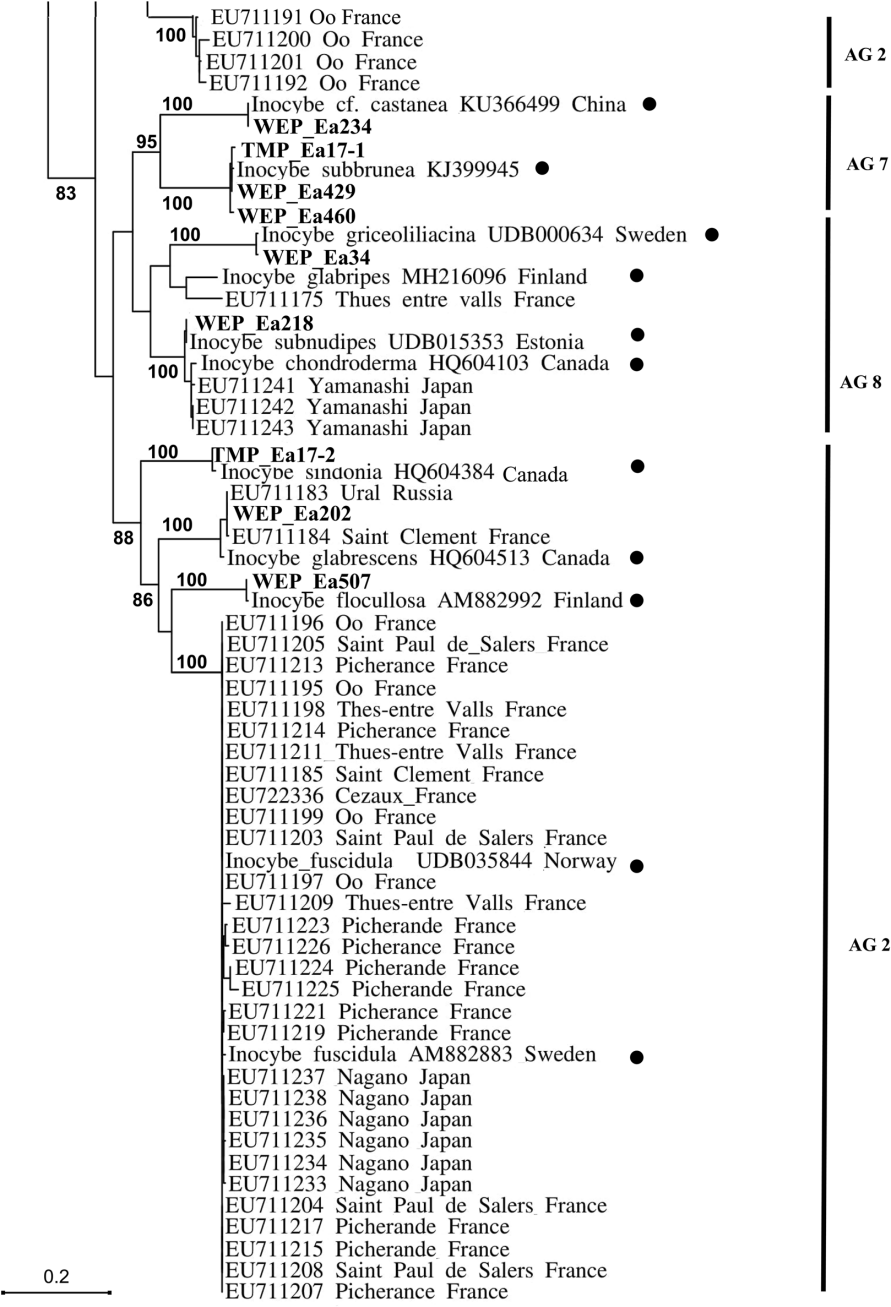
Phylogenetic analysis of *E. aphyllum* mycorrhizal fungi sampled for this study (bold) and from Roy et al. (2009b) as well as Liebel and Gebauer (2011) together with GenBank and UNITE sequences of fungi obtained from identified fruitbodies (black circle). The tree is based on a Maximum Likelihood analysis from an ITS alignment using model K81uf + I + G DNA (Posada and Crandal 1998). AG numbers on the side refer to alignment groups sensu Ryberg et al. (2008). Numbers indicate bootstrap values (based on 1,000 replicates) of branches, only bootstrap values > 80 are shown

In the monoclonal Wejherowo (WEP) population, fungal ITS was successfully identified from 40 plants (after cloning PCR products for 8 samples), leading to one *Inocybe* OTU per sample in all but one cloned PCR product (which displayed two *Inocybe* OTUs; Table S8). No rare *E. aphyllum* mycorhizal partner *i.e. Hebeloma, Lactarius* or *Thelephora*, were detected. Among the 13 different OTUs identified, the most abundant ones were related to *Inocybe terrigena* (32% of sequences), *I. leucoblema* (20%) and *I. mixtilis* (15%) (Table S8). *Inocybe* OTUs in WEP fell into six out of seven alignment groups found for *E. aphyllum* (AGs sensu Ryberg et al. 2008; Table S8, Figs. S6–S12). In comparison, the other 16 modestly (1–6 plants) sampled populations revealed in total 19 fungal OTUs including three *Hebeloma* species. Although only 6 OTUs were shared with WEP, the majority of them fell into the same AGs (2, 5, 8, and 16) thus representing closely related species (Figs. S6–S12). The phylogenetic breadth of fungal partner, in the unique WEP genetic individual with multiple ramets was comparable with the ones observed in the 29 plants sampled from 16 populations throughout the Eurasian range of the species (Fig. 5; Fig. S13). Thus, a single *E. aphyllum* genet, if sufficiently sampled, can encompass a large part of the phylogenetic diversity of fungal associates. The population from the Tatra Mountains (TMP), although not as intensively studied, showed the same pattern as WEP. In one of the large clones, 5 different OTUs were found among *Inocybe* and *Hebeloma*. Individuals forming mycorrhizae with both these genera were also found in SAN. At the opposite, genetically diverse individuals from OPP associated with the same genotype of *Hebeloma sinapizans*.

Finally, there was no correlation between fungal OTU phylogenetic distance and their hosts’ geographic distance either calculated in the whole data set (*r* = − 0.021, *p* = 0.36) or based on *Inocybe* data only (*r* = − 0.040, *p* = 0.35). It thus appears that the diversity of fungal associates is not distributed in a geographical pattern over the *E. aphyllum* range.

## Discussion

### Genetic diversity and clonality

Stolons and bulbils likely explain clonality within populations since no MLG covered two populations (markers are accurate) and the possibility of agamospermic (clonal) seed formation is negligible (Krawczyk et al. 2016). In *E. aphyllum*, stolon growth starts *ca*. 1–2 months before flowering time, even on non-flowering rhizomes (our pers. obs.), and continues until autumn. These observations are in line with overall low clonal diversity (*R*_*r*_ = 0.265). Large variation in clonal diversity between populations is most likely due to small and unequal sample size, which affect this metric. More samples, preferably in successive flowering years, are needed to properly assess the clonal diversity. At the extreme, the in-depth sampled WEP population harboured a single MLG over more than 3500 m^2^. This indicates that bulbils may travel faster than stolon growth, most probably due to water, animal disturbance or gravity after stolon decay, as reported by Robin (1999). Efficient asexual propagation may enable (i) effective exploitation of the patchy distribution of ectomycorrhizal fungi (Anderson et al. 2014; Genney et al. 2006), (ii) maintaining demographic stability after settlement by founders, (iii) avoidance of sexual costs, and (iv) existence of large MLGs.

High intrapopulation genetic diversity meets Hardy–Weinberg equilibrium, with no sign of linkage disequilibrium or inbreeding (*F*_IS_ = 0.094), despite frequently small population sizes. Thus, *E. aphyllum* appears to be mainly outcrossing, confirming the experimental pollinations by Krawczyk et al. (2016). Lack of relationship between *FIS*_*IS*_ and genotypic diversity in self-compatible clonal plant species was also confirmed in meta-analysis by Honnay and Jacquemyn (2008). In self-compatible plants, inbreeding depression may restrict recruitment of selfed progeny reducing the inbreeding coefficient (Husband and Shemske 1996; Lesica and Allendorf 1992) what may explain high mortality of seeds originating from experimental self-pollination in an in situ seed baiting experiment (Julita Minasiewicz, personal obs.). The high proportion of heterozygotes (H_o_ = 0.460, Table 2), high level of gene diversity (uH_e_ = 0.562), and homogeneity for these parameters among populations may seem surprising, since in often small *E. aphyllum* populations (Taylor and Roberts 2011) genetic drift and inbreeding are expected to reduce genetic variability and heterozygosity (Norman and Ellstrand 1993; Wright 1965). Yet, since studies of population genetics are typically biased toward large populations for statistical reasons, we lack data for naturally small and ephemeral populations (Shefferson et al. 2020). Relatively high levels of heterozygosity in *E. aphyllum* may be explained by the underestimation of population sizes due to a high dormancy. Because of the high cost of sprouting in non-photosynthetic plants, vegetative dormancy may be associated with lower mortality (Shefferson et al. 2011, 2018) or may represent a strategy to deal with spatiotemporal heterogeneities (Honnay and Bossuyt 2005), as dormant rhizomes may respond quicker to favourable conditions than seeds.

We suggest a balance between two mechanisms in population development. First, population founders arise from outcrossed seeds. A role of seed flow in colonisation is further supported by the occurrence of *E. aphyllum* in areas adjacent to existing or presumably extirpated ones (Nagy et al. 2018; Święczkowska 2010). Second, once a population is established, vegetative propagation maintains heterozygosity and limits genetic drift, assuming that some level of local sexual reproduction and gene flow alleviates the risk of monoclonal fixation (Balloux et al. 2003; Meloni et al. 2013). This may seem to be at odds with the common view that *E. aphyllum* populations are short-living (McCarthy 2010; Nagy et al. 2018; Robin 1999; Šegota and Alegro 2011; Söyrinki 1987). Yet, high levels of dormancy in MH plants (Roy et al. 2013; Shefferson et al. 2011, 2018) add complexity to patterns of shoot appearance from which the presence is inferred. Population continuity, when plants reappear after many years (McCarthy 2010; Tuulik et al. 2007), could only be verified by molecular methods, not available in the past. Uncertainties about the longevity of genets and the rate of dormancy preclude more direct conclusions. Complementary roles of seeds, in population founding and possibly gene flow, and clonal propagation explain our observations.

### Genetic structure and inferred gene flow

Our study documented a low genetic structure among Eurasian *E. aphyllum* populations, with evidence of admixture (Structure and PCA analyses) and low inter-population differentiation (*F*_ST_ = 0.106 for nuclear microsatellites and *F*_ST_ = 0.156 for plastid DNA when calculated on our reduced sample set; but even lower when pooling all samples into six geographical regions: *F*_*ST*_ = 0.047 and 0.112, respectively). However, Pyrenean and Massif Central populations showed the lowest genetic diversity, and the highest divergence supported by unique haplotypes and private alleles. This can be caused by distance to the main range, which entails higher genetic differentiation due to smaller effective population size and greater geographic isolation (Eckert et al. 2008; Samis et al. 2016) and/or alternatively by separate South-Western refugium during the Last Glacial Maximum (LGM), remaining ice-free and harbouring forest relicts (Lafontaine et al. 2014; Medail and Diadema 2009). However, there is rather a low potential for isolation of sub-populations and cryptic speciation in *E. aphyllum*, as shown by little (0.4%) variation in ITS sequence and the sharing of certain network-core nSSR haplotypes (H7, H20) among European populations.

The present data also show that, despite its MH lifestyle, *E. aphyllum* does not differ from autotrophic, allogamous orchids, which tend to show lower values of *F*_ST_ in comparison with other plant groups, pointing to a low overall differentiation and weak genetic structure (Phillips et al. 2012). This feature is usually associated with a high gene flow, likely resulting from the long-distance dispersal opportunities which are afforded by dust-like seeds of orchids (Arditti and Ghani 2000; Phillips et al. 2012) and/or preservation of ancestral polymorphism. Although the high rate of seed-mediated gene flow contrasts with some data showing a low fruiting success in *E. aphyllum* (Davies 2010; Geitler 1956; Reineke and Rietdorf 1998), a growing body of evidence rather indicates a variability of fruiting rate between sites (2–64%; Claessens and Klaynen 2005, 2011; Krawczyk 2016; Rakosy 2014; Ulrich 2008a, b a, b), probably resulting from variable pollinator abundance and competition with other plants for them (Claessens and Klaynen 2011; Rakosy 2014). A high number of seeds per capsule (4000–6000; Rakosy 2014) in comparison with other terrestrial orchids (Arditti and Ghani 2000), and their very small size in comparison with the majority of other European orchids (Mrkvicka 1994) may ensure efficient medium- to long-distance dispersal. Retention of ancestral polymorphism may also explain this pattern, but is less likely given the possible, albeit poorly shown, high turnover rate of populations. Alternatively, past population connectivity, for instance when a cooler climate enabled wider presence of the species over Europe, may explain haplotype sharing over disjunct parts of its present-day range. Supporting this hypothesis, the exceedingly rare plastid type H1 present only in southern Poland (OPP) in our study was also observed 1,800 km away to the north (Kem-Ludy Archipelago on the White Sea; GenBank accession KJ772292). Although homoplasy cannot be ruled out, this haplotype is distinguished by a specific mutation (24 bp deletion) in the *rps4* gene. Extensive sampling would help to elucidate more detailed patterns of gene flow in *E. aphyllum*.

### Mycorrhizal specificity

In the Wejherowo population (WEP), where 40 ramets sampled for mycorrhizal assessment represented the same genetic individual, *E. aphyllum* associated exclusively with species of the large, worldwide, ectomycorrhizal genus *Inocybe.* Our data as well as those of Roy et al. (2009b) and Liebel and Gebauer (2011) involve a subset of the *Inocybe* phylogenetic breadth, namely 17 OTU scattered through seven out of the 16 alignment groups (AGs) of this genus sensu Ryberg et al. (2008). However, there is a growing body of evidence that species of *Hebeloma,* although less frequently recruited than *Inocybe*, are also partners for *E. aphyllum*. In this context, the presence of even rarer partners of *E. aphyllum* like *Lactarius and Thelephora* (Roy et al. 2009b) or *Tomentellopsis* sp. (this study) still warrant analysis. On the one hand, targeting multiple species from one fungal lineage is a frequent feature of MH plants especially from temperate forests, *e.g.* in *Neottia nidus-avis* (Selosse et al. 2002), *Corallorhiza maculata* (Taylor et al. 2004), *Monotropa uniflora* (Bidartondo and Bruns 2005), and *Corallorhiza striata* (Barrett et al. 2010). On the other hand, narrow mycorrhizal specificity is not a prerequisite for mycoheterotrophy (*e.g.* Hynson and Bruns, 2009; Lee et al. 2015; Martos et al. 2009; Merckx et al. 2012; Roy et al. 2009a; Selosse et al. 2021). Lower than expected for typical temperate mycoheterotrophic plant, specificity towards fungal partner in *E. aphyllum* is emphasized by the fact that (1) *Hebeloma* is more distantly related to *Inocybe* than was previously thought (Matheny 2009), (2) as with *Inocybe*, *E. aphyllum* has the potential to track various species within *Hebeloma*, and (3) two subgenera of *Inocybe*, namely *Inosperma* (AG 3) and *Mallocybe* (AG 16), were elevated to generic rank based on their genetic divergence (Matheny et al. 2020).

*E. aphyllum* associations revealed no clear pattern of geographical specialisation that would fit a geographic mosaic of coevolution with fungi (Thompson 2005). First, different ramets belonging to the same genetic individual (clones) can associate with various species of *Inocybe* and *Hebeloma* (e.g. at WEP and TMP). In WEP, a single clone harboured 68% of *Inocybe* OTUs known to be associated with *E. aphyllum,* distributed in six AGs sensu Ryberg. The small portion of the population as well as small rhizome part we were authorised to sample at WEP likely makes this value underestimated. Second, a single rhizome can be mycorrhizal with several *Inocybe* OTUs (see Roy et al. 2009b). Since most of the *Inocybe* phylogenetic diversity can thus associate with a single genotype, host genotype control over partner selection seems negligible. Third, various genotypes of *E. aphyllum* can associate with a single genotype of *Hebeloma* (e.g. at OPP). Finally, partner identity showed no geographic structuring over the Eurasian populations observed, strongly arguing against a geographical mosaic of association, despite our large sampling range (Fig. 2).

Explanation of the absence of mosaicism may lie with the preferential allogamous habit of *E. aphyllum* and the high dispersal capabilities discussed above, which tend to promote generalism (Poisot et al. 2011). The ectomycorrhizal forest habitat of *E. aphyllum* and its mycorrhizal fungi range continuously over Eurasia and mainly over Europe, without geographic barriers to species and gene flow (as stated for fungal species; Roy et al. 2008; Vincenot et al. 2012), which can favour genetic homogenisation. Here, we believe that variation in the fungal partners of *E. aphyllum* results rather from the local availability of *Inocybe* and *Hebeloma* species, and possibly of other ectomycorrhizal fungi rarely associated with the species.

In the future, both the Asian origin of *E. aphyllum* and the absence of local specificities await further confirmation with more samples from the eastern part of the species range. In addition, as for most orchids, a better understanding of the effective range of seed and pollen dispersal is pending, to confirm that the absence of differentiation observed can be due to gene flow.

## Supplementary Information

Below is the link to the electronic supplementary material.

Supplementary file1 (PDF 2801 KB)

Supplementary file2 (PDF 502 KB)

Supplementary file3 (XLSX 143 KB)
