# Supplementary material for: Weak population spatial genetic structure and low infraspecific specificity for fungal partners in the rare mycoheterotrophic orchid Epipogium aphyllum
Source: J Plant Res. 2022 Jan 6;135(2):275–93. doi: 10.1007/s10265-021-01364-7 (PMC8894228; doi:10.1007/s10265-021-01364-7)
Supplement: Supplementary file 1 — Supplementary file1 (PDF 2801 KB) [file 10265_2021_1364_MOESM1_ESM.pdf]

Journal of Plant Research

WEAK POPULATION SPATIAL GENETIC STRUCTURE AND LOW INFRASPECIFIC SPECIFICITY FOR FUNGAL PARTNERS IN THE RARE MYCOHETEROTROPHIC ORCHID *EPIPOGIUM APHYLLUM*

Julita Minasiewicz[1*], Emilia Krawczyk[1], Joanna Znaniecka[2], Lucie Vincenot[3], Ekaterina Zheleznaya[4], Joanna Korybut-Orlowska[1], Tiiu Kull[5], Marc-André Selosse[1,6]

[1] *University of Gdańsk, Faculty of Biology, Department of Plant Taxonomy and Nature Conservation, ul. Wita Stwosza 59, 80-308 Gdańsk, Poland;* [2] *Intercollegiate Faculty of Biotechnology of University of Gdansk and Medical University of Gdansk, Abrahama 58, 80-307 Gdansk, Poland;* [3] *Normandie University, UNIROUEN, INRAE, ECODIV, 76000 Rouen, France;* [4] *Peoples' Friendship University of Russia, Podolskoye shosse 8/5,115093, Moscow Russia. Timiryazev State Biological Museum, Malaya Gruzinskaya, 15, 123242, Moscow, Russia;* [5] *Estonian University of Life Sciences, Tartu, Estonia;* [6] *Institut de Systématique, Evolution, Biodiversité (ISYEB), Muséum National d'Histoire Naturelle, CNRS, Sorbonne Université, EPHE, CP 39, 57 rue Cuvier, 75005 Paris, France.*

* Author for correspondence: e-mail: julita.minasiewicz@ug.edu.pl

Figure S1: Phylogenetic clustering of *E. aphyllum* on ITS alignment;

Figure S2: Genotype accumulation curve for 248 samples of *Epipogium aphyllum* over 9 loci.

Figure S3: Determination of most supported K value based on STRUCTURE software results with the Evanno method;

Figure S4: Geographical distribution of the two clusters found with the software STRUCTURE;

Figure S5: A Mantel test for isolation by distance based on the 82 *E. aphyllum* MLGs - a scatter plot;

Figure S6-S12: Phylogenetic alignments between *Inocybe* OTUs found in rhizomes of *E. aphyllum* in the present study and Roy *et al*. (2009*b*) and *Inocybe* sp. sequences included in alignment groups by Ryberg *et. al*. (2008). Alignment Groups: 2, 3, 5, 7, 8, 13, 16;

Figure S13: Frequency distribution of *Inocybe* OTUs cumulating this study and Roy *et al*. (2009*b*) over alignment groups *sensu* Ryberg *et al*. (2008);

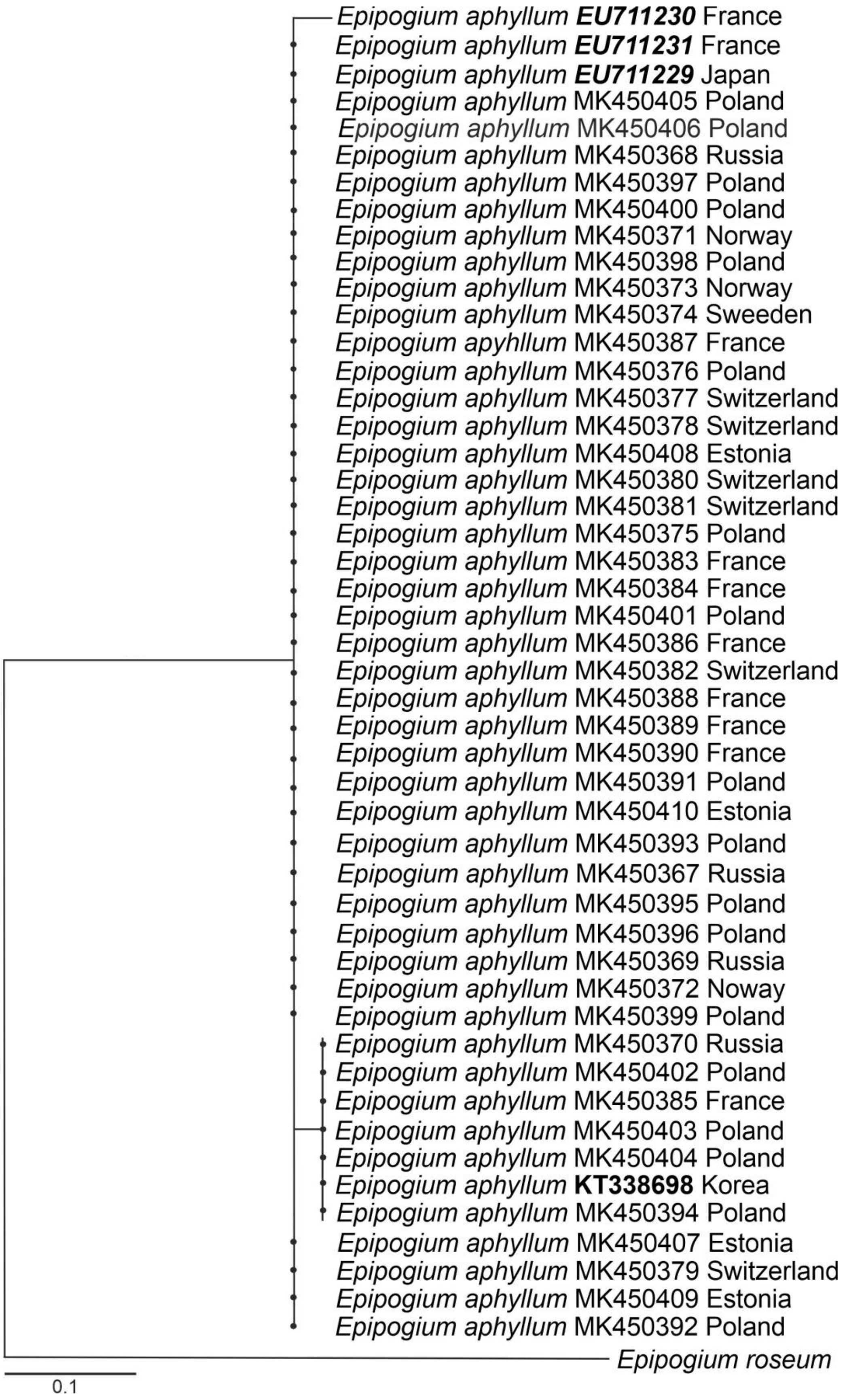

Figure S1. Clustering of *E. aphyllum* from studied populations and selected sequences from GenBank (bolded) with respect to sequences of *E. roseum* (EU11232), as an outgroup (ML on alignment of ITS; GTR model, bootstrap with 1000 repetitions, all values below 80).

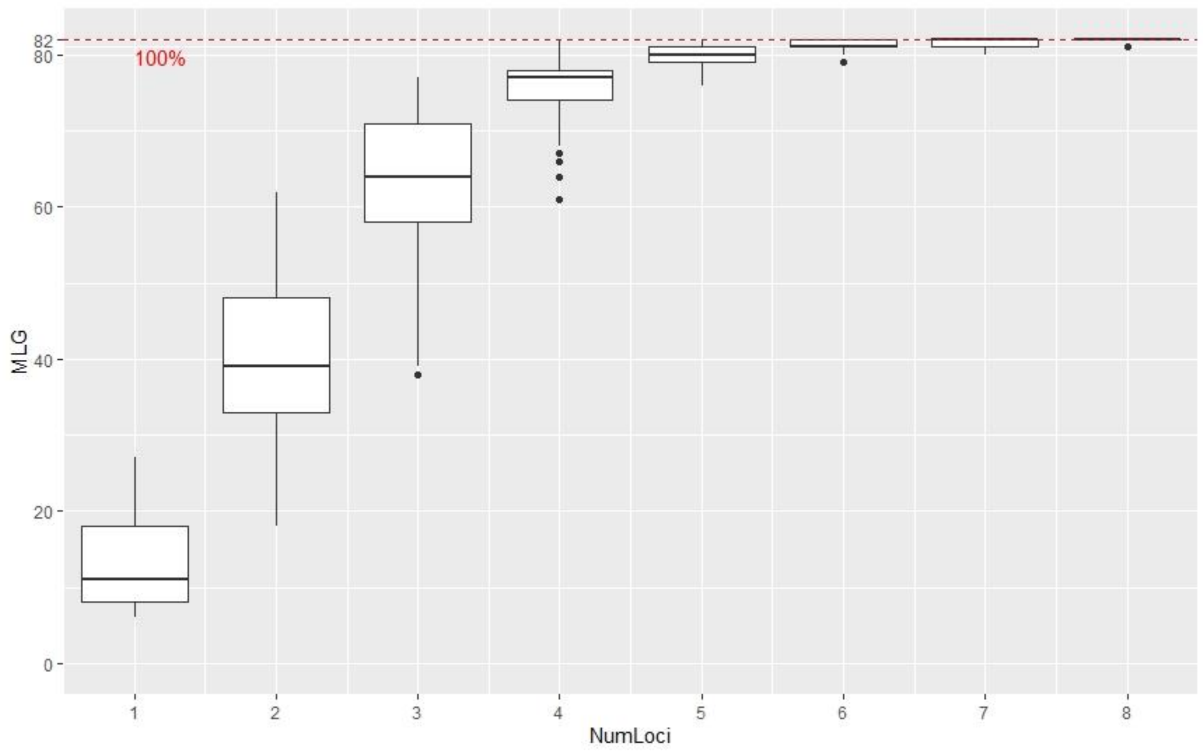

Figure S2. Genotype accumulation curve for 248 samples of *Epipogium aphyllum* over 9 loci.

The horizontal axis represents the number of loci randomly sampled without replacement up to n-1 loci. The vertical axis represents number of unique multilocus genotypes (MLG) observed (n=82) in the dataset. Applied set of loci sufficiently resolve unique genotypes as accumulation curve reach plateau with 9 loci

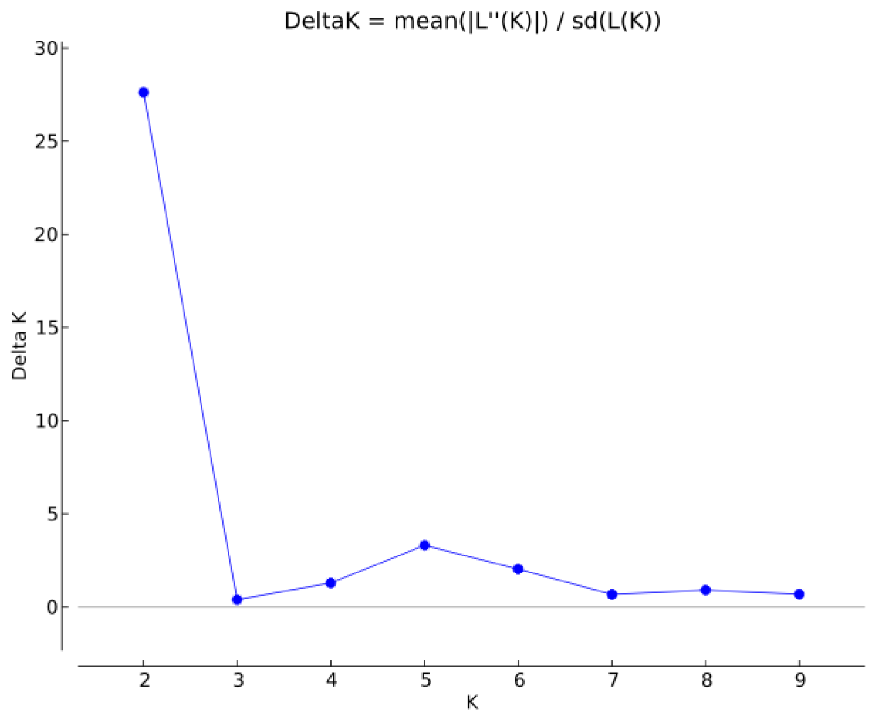

Figure S3. Determination of most supported K value (number of genetic clusters for the complete sample set of 82 MLGs of *E. aphyllum*), based on STRUCTURE software results with the Evanno method.

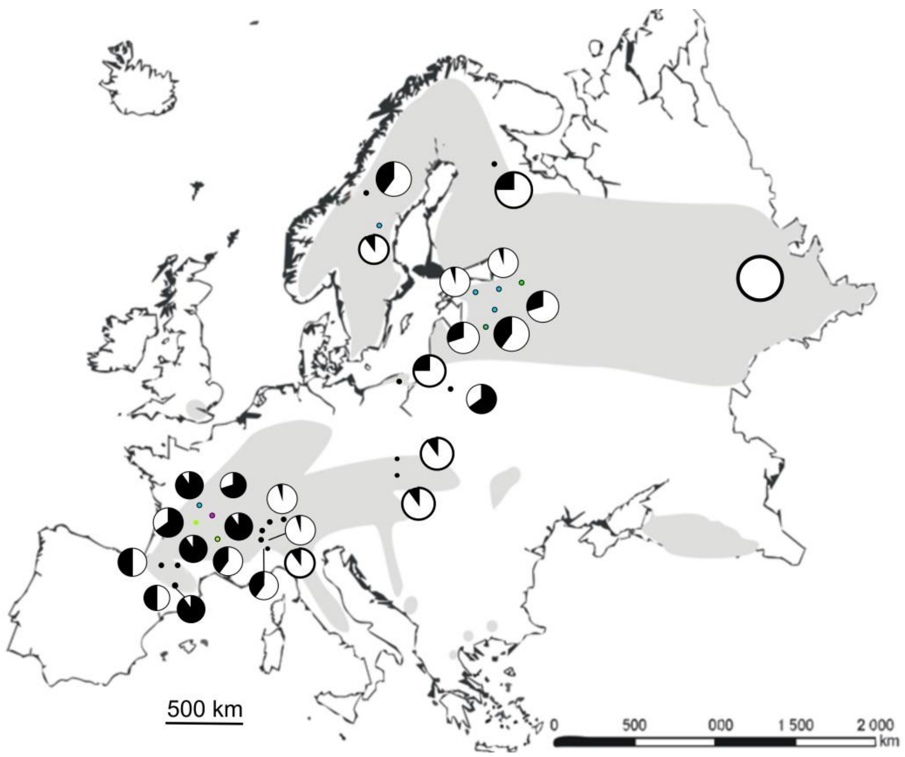

Figure S4. Geographical distribution of the two clusters found with the software STRUCTURE (see Figure 3) among the 27 *E. aphyllum* populations sampled. Their geographical region and position in the range of the species are given above the figure A. Note that the map is distorted to limit the space between Europe and the easternmost population.

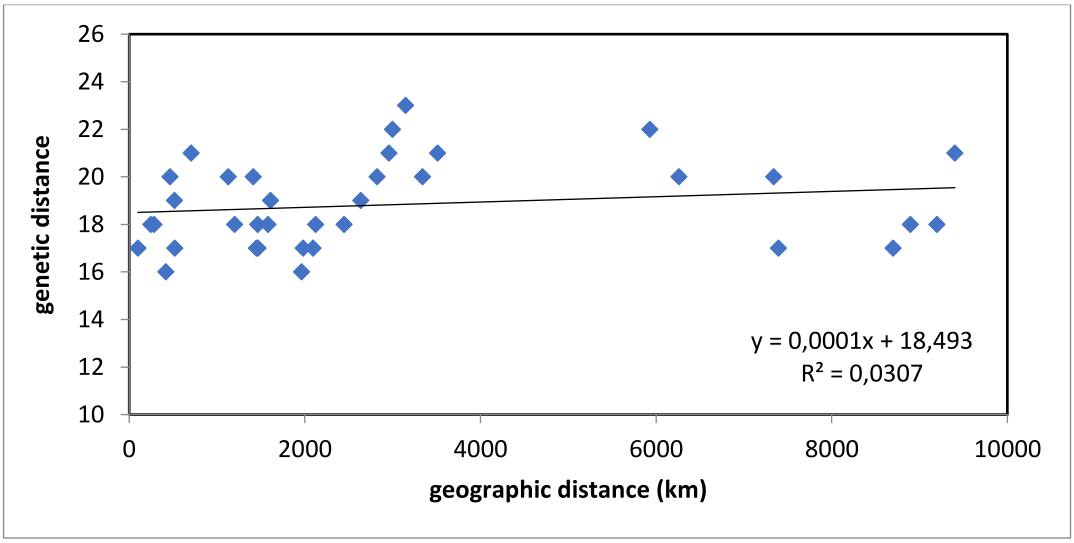

Figure S5. Mantel test of isolation by distance for 9 populations of *E. aphyllum*. Scatter plot displays geographic *versus* genetic distance.

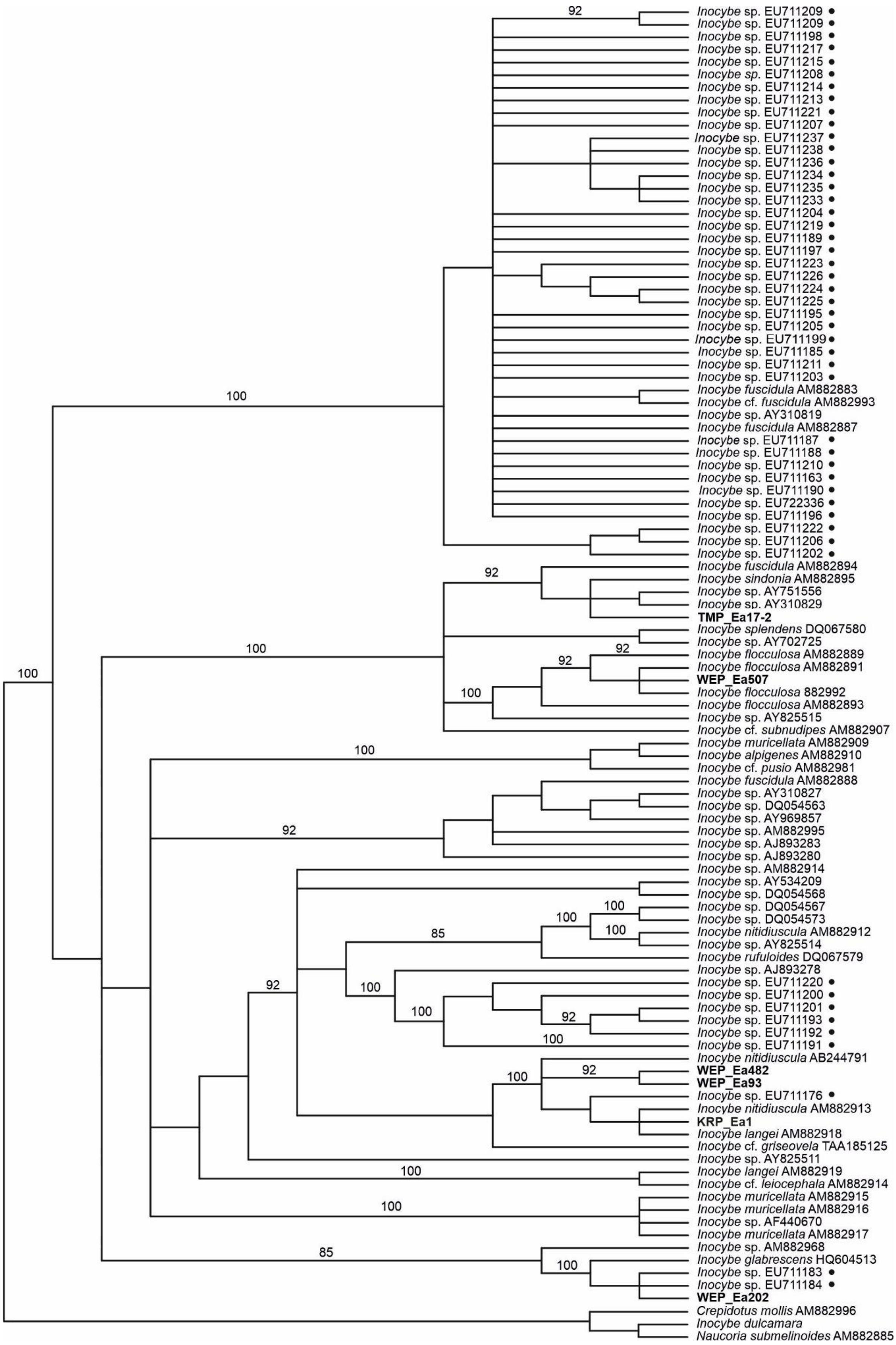

Figure S6. Alignment Group 2 *sensu* Ryberg.
Phylogenetic alignment between *Inocybe* OUT found in rhizomes of *E. aphyllum* in the present study (bolded) and Roy *et al.* (2009, dots) and *Inocybe* sp. sequences from GenBank compiled in smaller alignment groups as described in Ryberg *et al*. (2008). (Strict consensus tree on alignment of ITS; parsimony criterion with TBR swapping, jackknife with 1000 repetitions).

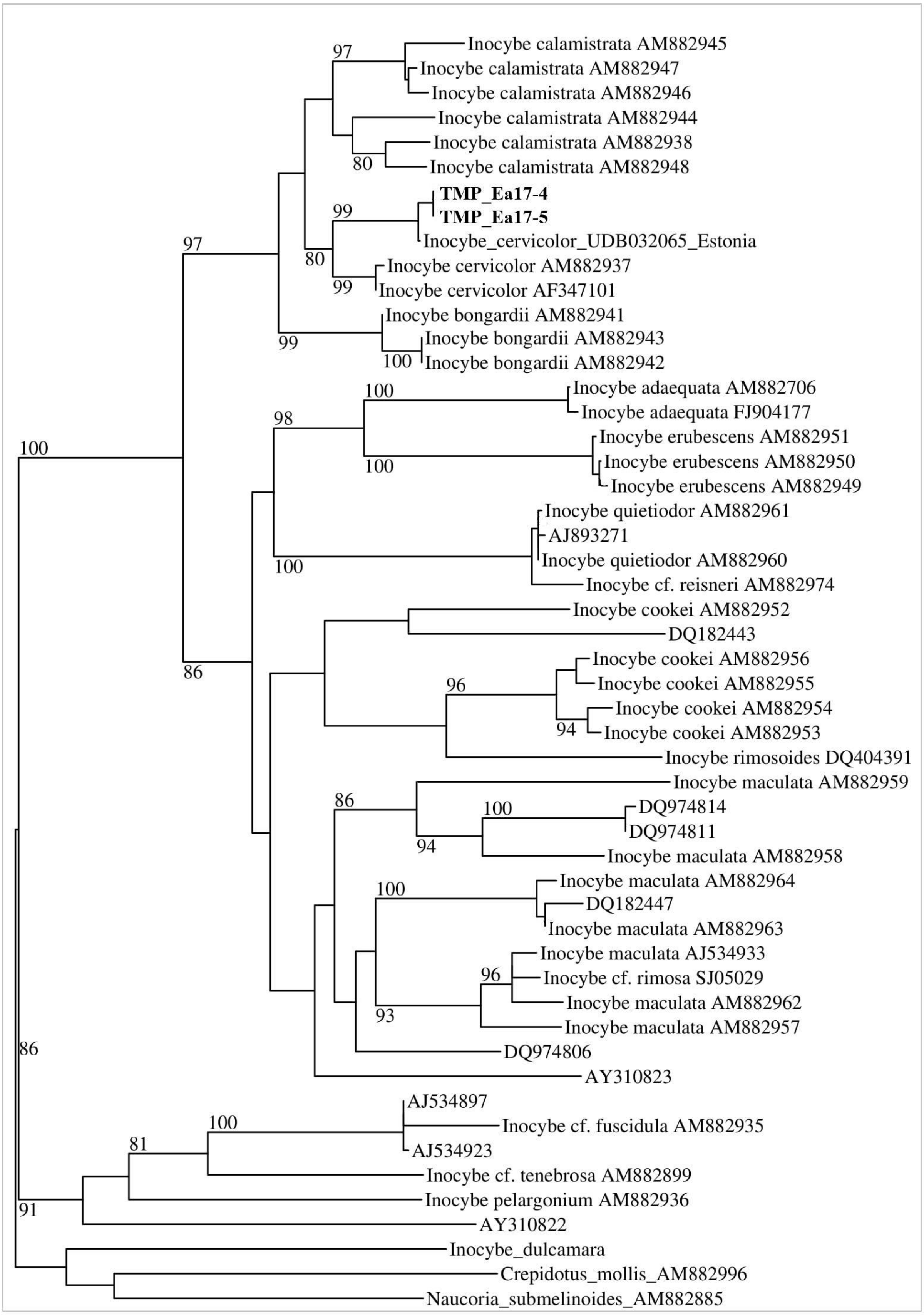

Figure S7. Alignment Group 3 *sensu* Ryberg.
Phylogenetic alignment between *Inocybe* OUT found in rhizomes of *E. aphyllum* in the present study (bolded) and *Inocybe* sp. sequences from GenBank compiled in smaller alignment groups as described in Ryberg *et al*. (2008). (Strict consensus tree on alignment of ITS; parsimony criterion with TBR swapping, jackknife with 1000 repetitions).

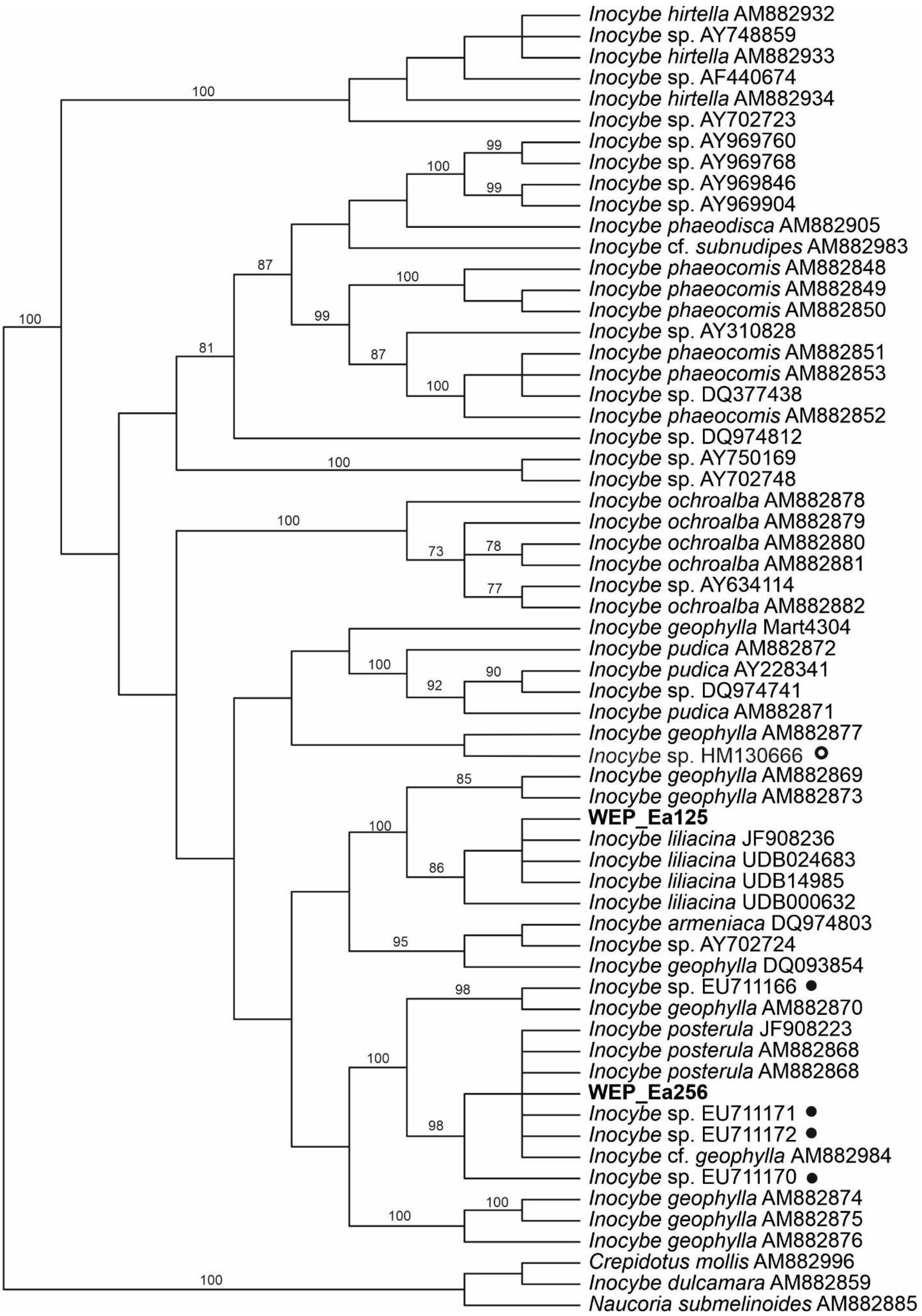

Figure S8. Alignment Group 5 *sensu* Ryberg.
Phylogenetic alignment between *Inocybe* OUT found in rhizomes of *E. aphyllum* in the present study (bolded), Roy *et al*. (2009, black dots), Liebel & Gebauer (2011; white dot) and *Inocybe* sp. sequences from GenBank compiled in smaller alignment groups as described in Ryberg *et al*. (2008). (Strict consensus tree on alignment of ITS; parsimony criterion with TBR swapping, jackknife with 1000 repetitions).

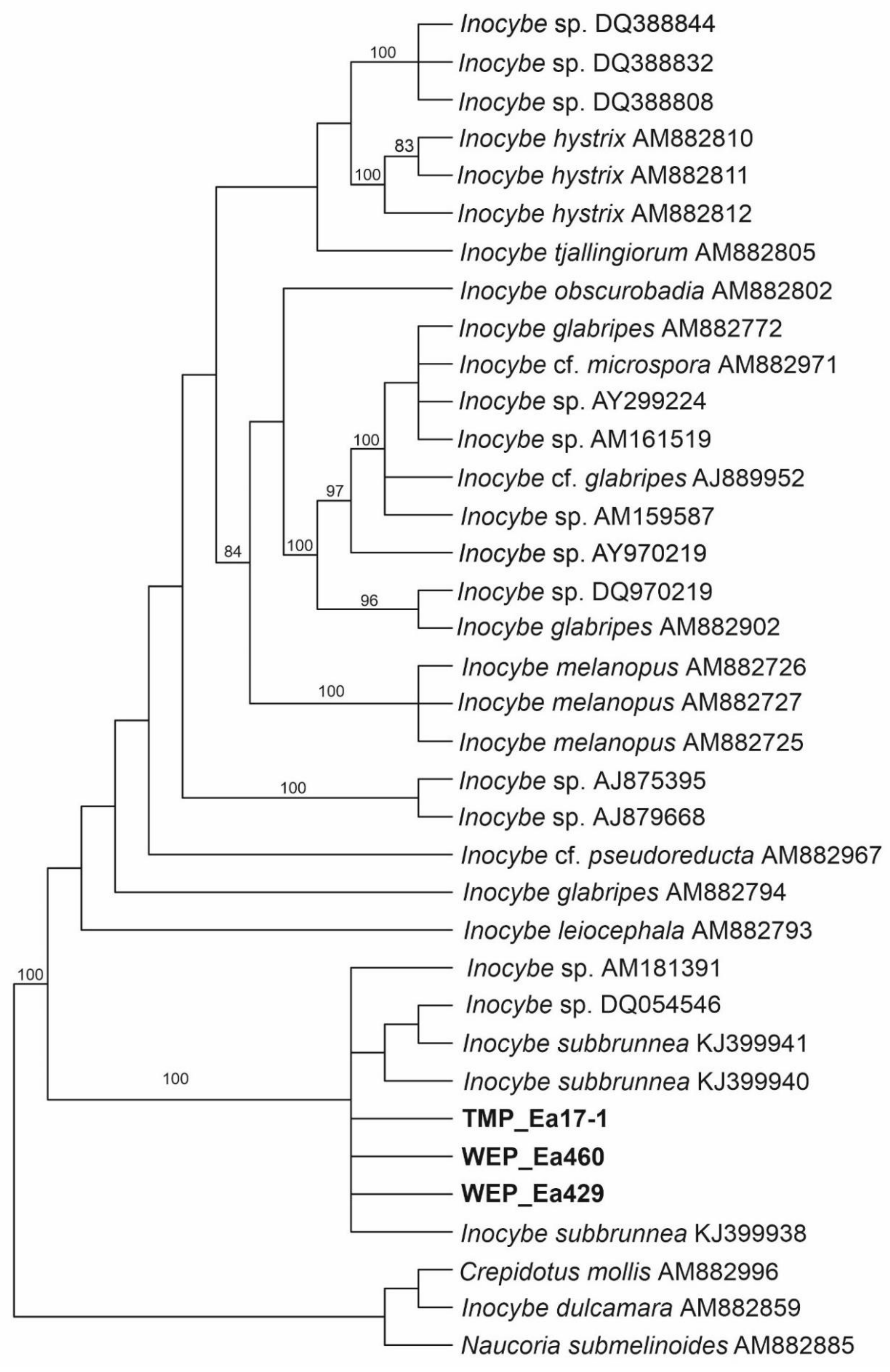

Figure S9. Alignment Group 7 *sensu* Ryberg.
Phylogenetic alignment between *Inocybe* OUT found in rhizomes of *E. aphyllum* in the present study (bolded) and *Inocybe* sp. sequences from GenBank compiled in smaller alignment groups as described in Ryberg *et al*. (2008). (Strict consensus tree on alignment of ITS; parsimony criterion with TBR swapping, jackknife with 1000 repetitions).

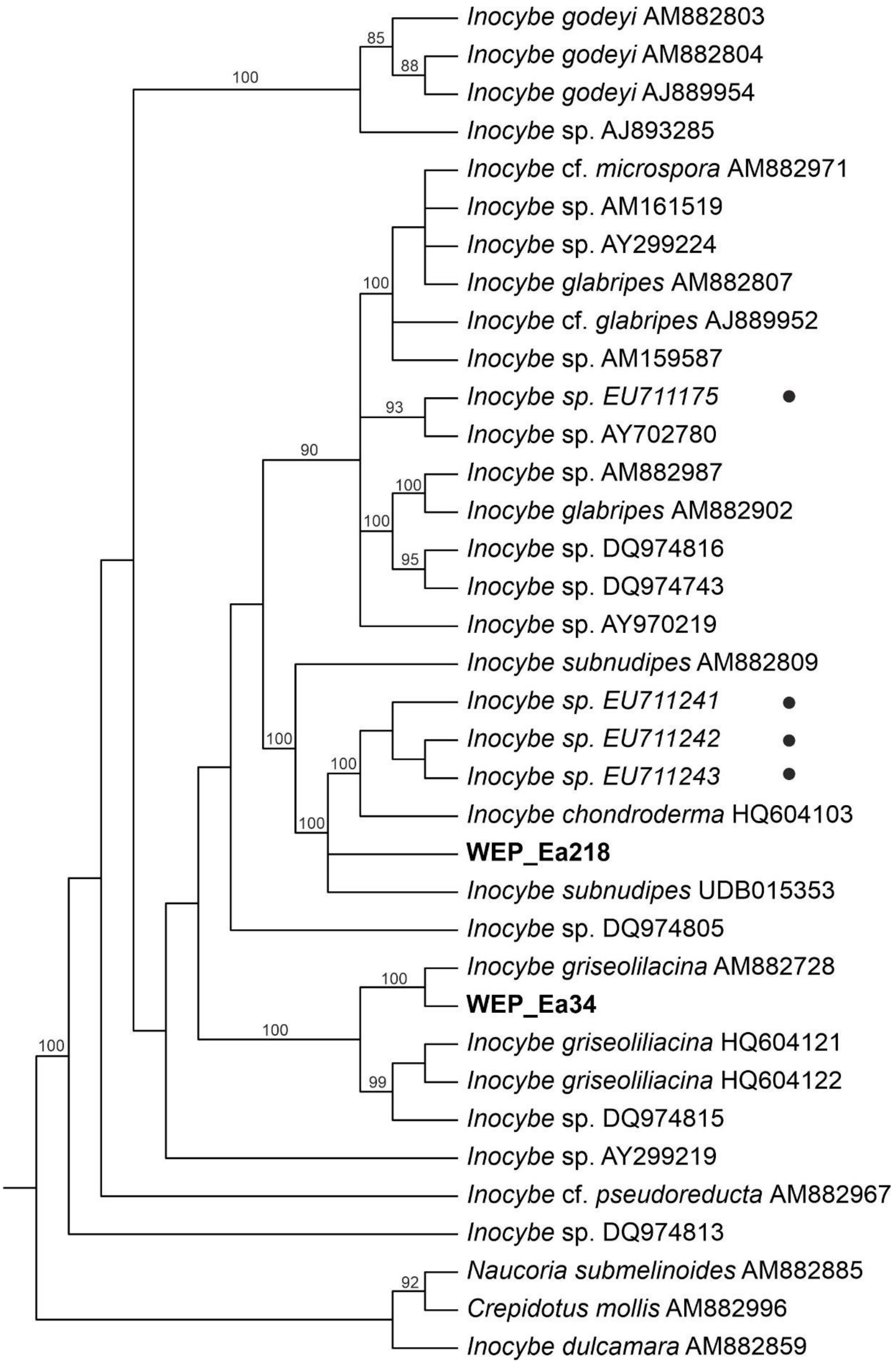

Figure S10. Alignment Group 8 *sensu* Ryberg.
Phylogenetic alignment between *Inocybe* OUT found in rhizomes of *E. aphyllum* in the present study (bolded) and Roy *et al*. (2009, dots) and *Inocybe* sp. sequences from GenBank compiled in smaller alignment groups as described in Ryberg *et al*. (2008). (Strict consensus tree on alignment of ITS; parsimony criterion with TBR swapping, jackknife with 1000 repetitions).

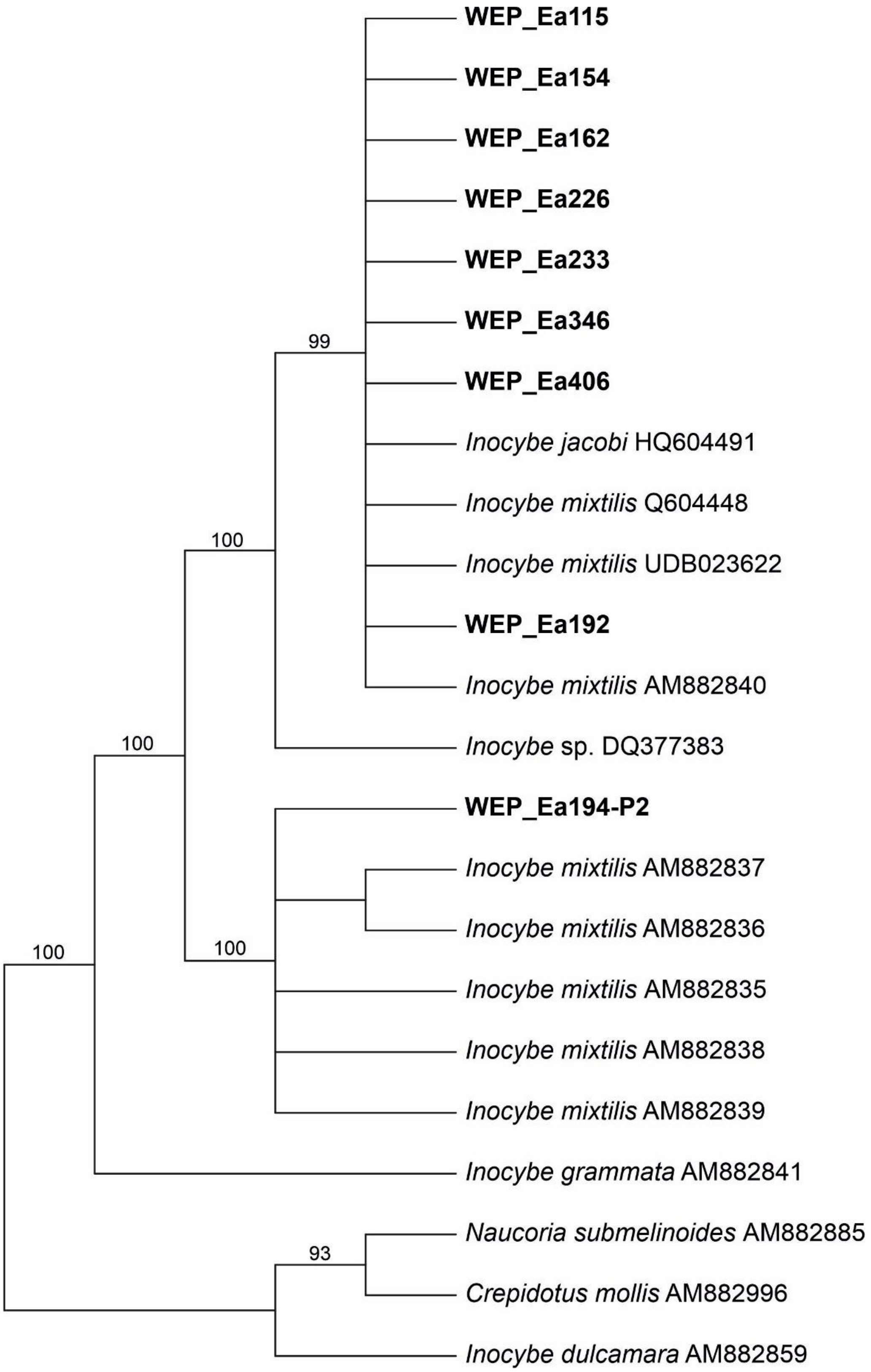

Figure S11. Alignment Group 13 *sensu* Ryberg.
Phylogenetic alignment between *Inocybe* OUT found in rhizomes of *E. aphyllum* in the present study (bolded) and *Inocybe* sp. sequences from GenBank compiled in smaller alignment groups as described in Ryberg *et al*. (2008). (Strict consensus tree on alignment of ITS; parsimony criterion with TBR swapping, jackknife with 1000 repetitions).

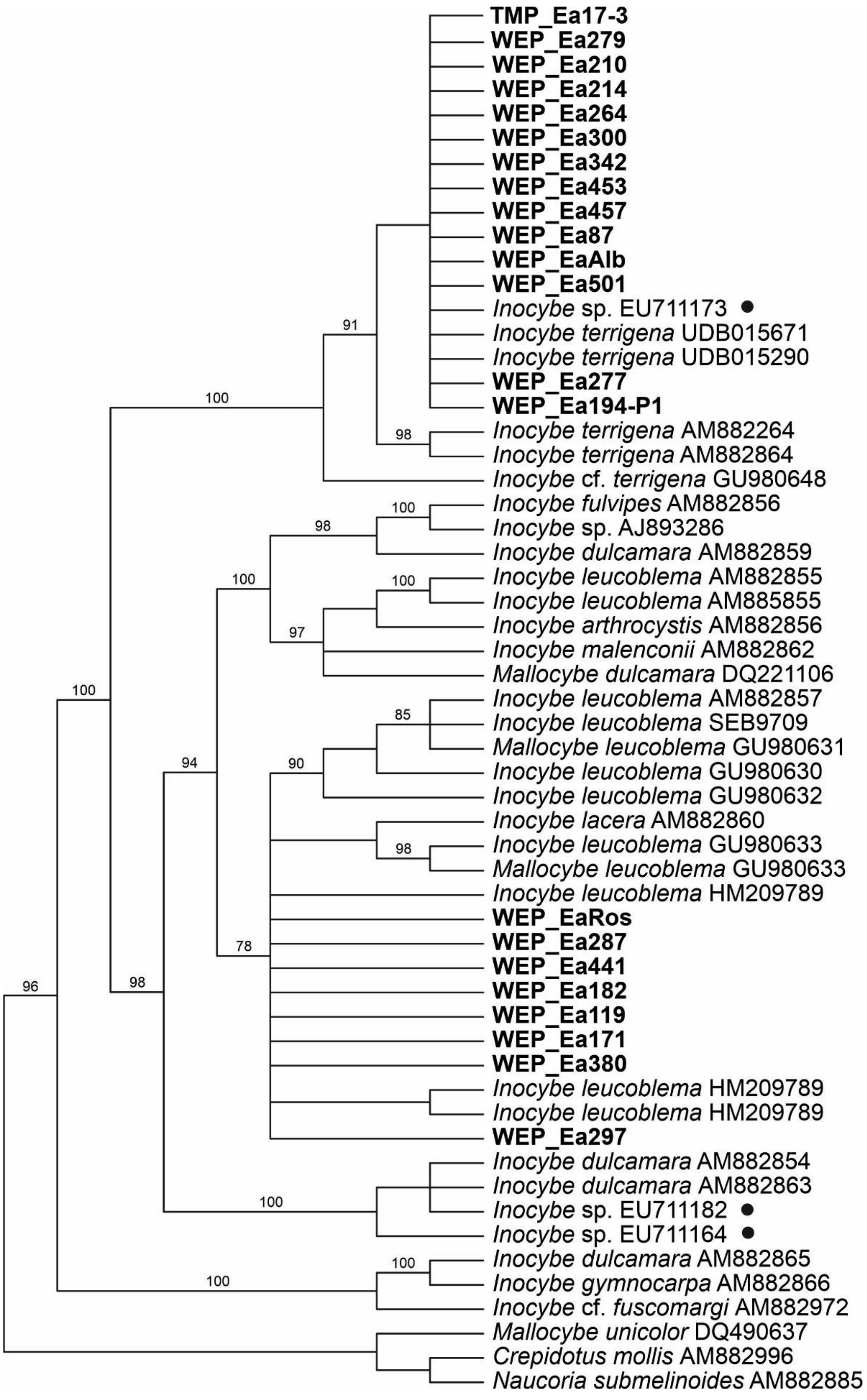

Figure S12. Alignment Group 16 *sensu* Ryberg.
Phylogenetic alignment between *Inocybe* OUT found in rhizomes of *E. aphyllum* in the present study (bolded) and Roy *et al.* (2009, dots) and *Inocybe* sp. sequences from GenBank compiled in smaller alignment groups as described in Ryberg *et al*. (2008). (Strict consensus tree on alignment of ITS; parsimony criterion with TBR swapping, jackknife with 1000 repetitions).

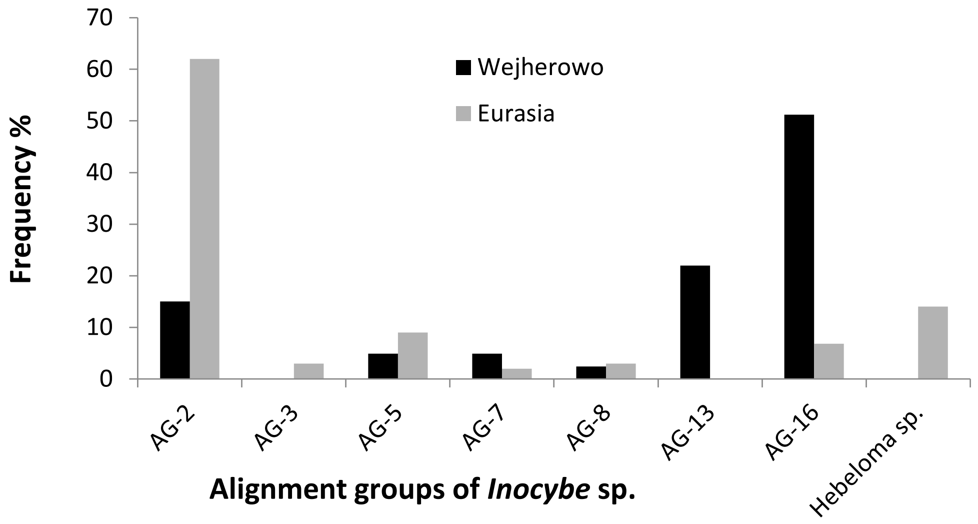

Figure S13. Frequency distribution of *Inocybe* sp. and *Hebeloma* sp. in the 40 *Epipogium aphyllum* samples from Wejherowo population (this study) and the 58 samples from 16 populations sampled throughout its Eurasiatic range, cumulating this study, Roy *et al*. (2009*b*) and Liebel and Gebauer (2011).
