# Supplementary material for: Weak population spatial genetic structure and low infraspecific specificity for fungal partners in the rare mycoheterotrophic orchid Epipogium aphyllum
Source: J Plant Res. 2022 Jan 6;135(2):275–93. doi: 10.1007/s10265-021-01364-7 (PMC8894228; doi:10.1007/s10265-021-01364-7)
Supplement: Supplementary file 2 — Supplementary file2 (PDF 502 KB) [file 10265_2021_1364_MOESM2_ESM.pdf]

Journal of Plant Research

WEAK POPULATION SPATIAL GENETIC STRUCTURE AND LOW INFRASPECIFIC SPECIFICITY FOR FUNGAL PARTNERS IN THE RARE MYCOHETEROTROPHIC ORCHID *EPIPOGIUM APHYLLUM*

Julita Minasiewicz[1*], Emilia Krawczyk[1], Joanna Znaniecka[2], Lucie Vincenot[3], Ekaterina Zheleznaya[4], Joanna Korybut-Orlowska[1], Tiiu Kull[5], Marc-André Selosse[1,6]

[1] *University of Gdańsk, Faculty of Biology, Department of Plant Taxonomy and Nature Conservation, ul. Wita Stwosza 59, 80-308 Gdańsk, Poland;* [2] *Intercollegiate Faculty of Biotechnology of University of Gdansk and Medical University of Gdansk, Abrahama 58, 80-307 Gdansk, Poland;* [3] *Normandie University, UNIROUEN, INRAE, ECODIV, 76000 Rouen, France;* [4] *Peoples' Friendship University of Russia, Podolskoye shosse 8/5,115093, Moscow Russia. Timiryazev State Biological Museum, Malaya Gruzinskaya, 15, 123242, Moscow, Russia;* [5] *Estonian University of Life Sciences, Tartu, Estonia;* [6] *Institut de Systématique, Evolution, Biodiversité (ISYEB), Muséum National d'Histoire Naturelle, CNRS, Sorbonne Université, EPHE, CP 39, 57 rue Cuvier, 75005 Paris, France.*

* Author for correspondence: e-mail: julita.minasiewicz@ug.edu.pl

Table S1: Details of sampling locations and effort;

Table S2: Details of nSSR loci applied;

Table S3: Details of plastid DNA loci applied;

Table S4: PCR conditions for plastid DNA loci amplification;

Table S5: A description of the 22 plastid DNA haplotypes;

Table S6: Pairwise F_{ST} for nine populations of *E. aphyllum* based on nuclear SSR and plastid DNA;

Table S7: Pairwise F_{ST} on a regional level of *E. aphyllum* nuclear SSR and plastid DNA diversity;

Table S8: Characterisation of symbiotic fungal OTUs of *E. aphyllum* found in the present study.

Table S1. Details of sampling locations and sampling effort of the 27 populations of *E. aphyllum*; N_{ITS} - population sample sizes for ribosomal DNA internal transcribed spacer diversity; N_{DNA} - population sample sizes for cpDNA diversity; N_{HAP} – number of haplotypes after correction for clones; N_{SSR} – population sample size for nuclear microsatellite diversity; MLG - number of multilocus genotypes observed in population after correction for clones; eMLG – number of expected MLG at the smallest sample size ≥ 10 based on rarefaction; *R* – genotypic richness; R_r – genotypic richness in population ≥ 10 based on rarefaction; *na* – not assessed. In bold, populations ≥ 4 MLG for which population genetic analyses have been conducted.

Location in range	Region	Code	Location of population	Lat/long (N/E)	N_{ITS}	N_{DNA}	N_{HAP}	N_{SSR}	MLG (eMLG)	*R* (R_r)
**WESTERN**	**Pyrenees**	**TVF**	**Thuès-entre-Valls**	42.515/2.252	-	3	3	4	4	1.000
		CXF	Cazaux	43.052/1.509	-	2	1	2	1	*na*
		OOF	Oô	42.796/0.505	-	2	1	2	1	*na*
	**Massif Central**	SCF	Saint-Clément	44.924/2.661	3	1	1	5	3	0.500
		BGF	Bois de Grandval	45.032/2.769	-	7	3	7	3	0.333
		**MOF**	**Malbo**	45.000/2.734	2	21	5	26	5 (3.09)	0.154 (0.084)
		SSF	Saint-Paul-de-Salers	45.140/2.517	-	1	1	1	1	*na*
		PSF	Ponteils	44.891/3.958	1	-	-	2	2	*na*
		PEF	Picherande	45.463/2.769	1	3	1	3	1	*na*
**SOUTHERN**	**Alps**	**BNF**	**Boscodon**	44.494/6.453	-	4	4	4	4	1.000
		BRF	Brizon	46.036/6.440	-	-	-	2	1	*na*
		LVF	Lans en Vercors	45.129/5.588	1	1	1	2	2	*na*
		VLF	Villard-de-Lans	45.069/5.550	-	1	1	1	1	*na*
		**VUS**	**Val d'Uina**	46.608/7.121	6	6	6	6	6	1.000
	**Carpa-thians**	**OPP**	**Ojcowski National Park**	50.185/19.817	11	25	11	32	11 (11.00)	0.322 (0.322)
		**TMP**	**Tatra Mountains**	49.255/19.897	5	26	8	35	9 (8.49)	0.235 (0.220)
**NORTHERN**	**Baltic region**	WEP	Wejherowo	54.624/18.212	1	32	1	80	1 (1)	0.000 (0.000)
		KRP	Kozi Rynek	53.799/23.227	1	1	1	1	1	*na*
		SAN	Snåsa	64.200/12.200	3	2	2	4	3	0.666
		RKS!	Ragunda Kommun	63.100/16.300	1	1	1	1	1	*na*
		HIE	Hiiumaa Island	58.933/24.241	1	1	1	1	1	*na*
		JAE	Jouga	59.176/27.465	1	1	1	1	1	*na*
		KEE	Kose	59.329/27.499	1	1	1	1	1	*na*
		**JEE**	**Jänioe**	59.264/25.518	1	2	2	7	5	0.666
		**LDR**	**Luzhsky District**	58.983/29.688	-	4	4	4	4	1.000
		VRR	Vologda Region	59.693/37.911	2	3	1	4	1	0.250
**EAST ERN**	**Asia**	**ZDR**	**Zeysky District**	54.085/126.87 4	2	7	7	10	8 (8.00)	0.778 (0.778)
				**TOTAL**	**44**	**158**	**69**	**248**	**82**	(0.265)

! – sample from Jodrell Kew DNA bank (accession no. 19248)

Table S2. Microsatellite loci for *E. aphyllum*, with repeated motif, primer sequences (5'-3' sequence), amplicon size range, and PCR annealing temperatures (T_m).

Marker name	Motif	Forward primer	Reverse primer	Size range (bp)	No of alleles	T_m
Epi_8	$(AAG)_5$	AACCCTTTAAGCCAGTGGGA	TCAACCCGTCTTGTTCAGGT	207-213	3	60
Epi_13	$(AG)_{13}$	TTCGATCGATCACCAACCTAA	CCTCTTGAAGGCTTTGAAGGTT	214-244	8	64
Epi_17	$(AG)_9$	GAATGTCTTCCCATTCGACG	AGTCGTGCATGTTTGACCCT	177-191	6	63
Epi_21	$(AT)_8$	ACGATGCACTTGAGACCTGA	CGAGGGTTATTAAGGCTTGATTATT	185-191	4	58
Epi_22	$(AAG)_9$	AGTATGGCGCTTTCGCTTC	CTCCGGAGCAGGATGAAATA	139-163	8	60
Epi_26	$(TC)_7C(TC)_2$	TGTTGAAGAACCTGTGGACTAAA	TATCCCATTGTTCGGACCAG	313-317	3	58
Epi_45	$(AG)_{11}$	CCGATCAACGAATGGACAA	TGGTCTTCATCTCTACAGTCT	183-211	13	60
Epi_49 [a]	$(AT)_{11}$	AGGGAGGCGCATATCAATTA	GCCCACGTATGAGGGTAAAA	189-205	9	60
Epi_51	$(AG)_{14}$	GAAGACTTTGACTGTTCGTTCC	ACACCTGATTGCTCCCAGTT	(218)226-238	7	60
Epi_53 [a]	$(AT)_{13}$	CAAATGGAAGAGCCAGGTGA	TCCCTAACAAGCTTTCCCAG	253-273	10	60
Epi_55 [a]	$(AG)_{11}$	TGTTCTCCCTCTATCTCCCG	AGGGCTCTGAGAAACCCTTG	188-210	10	60
Epi_60 [b]	$(AT)_{12}$	ATTGAGCAAAGCAGATGGCT	CAATGCCCGATCAGTTAAGC	146-152	4	58
Epi_61	$(AAT)_{11}$	ATTAACGGGTGAGGTGATCG	CCAGCAAGAAAGGAGTGTCC	205-214	4	60

[a] discarded because of evidence of null alleles.
[b] discarded due to missing data

Table S3. Plastid loci for *E. aphyllum*, with primer names and sequences.

	Locus	Foreward primer	Reverse primer
1	*accd*-*trn*E(UUC)	***accd*-5447F:** AATTCTTCATTTTCTTCAGTACTACCA	***trn*E-5989R:** ACTGGGTTGAGCTGGATTTG
2	*rps4*	***rps4*-1F:** CGAAGTTTGTCCGCTCTACC	***rps4*-1R** ATTCGACAACCAATAGCTCA
3	*rps12*-*trn*L(GCA)	***rps12*-1F:** AGAGTGTATGTGCGATTCGTTT	***trnL*-1R:** CATCTGGATTTGAACCAGAGTAT

Table S4. PCR conditions for plastid loci amplification.

<table>
<tr><th></th><th>Locus</th><th>Initial de-naturation</th><th>Denaturation</th><th>Annealing</th><th>Extension</th><th>Final Extension</th></tr>
<tr><td rowspan="2">1</td><td rowspan="2">accd-trnE(UUC)</td><td rowspan="2">96 °C for 5 min</td><td>95°C for 30 s</td><td>58.5 °C for 30 s</td><td>72 °C for 120 s</td><td rowspan="2">72 °C for 10 min</td></tr>
<tr><td colspan="3">30 cycles</td></tr>
<tr><td rowspan="2">2</td><td rowspan="2">rps4</td><td rowspan="2">96 °C for 5 min</td><td>95°C for 30 s</td><td>58.5 °C for 30 s</td><td>72 °C for 120 s</td><td rowspan="2">72 °C for 10 min</td></tr>
<tr><td colspan="3">30 cycles</td></tr>
<tr><td rowspan="4">3</td><td rowspan="4">rps12-trnL(GCA)</td><td rowspan="4">96 °C for 5 min</td><td>95°C for 30 s</td><td>60 °C for 30 s</td><td>72 °C for 45 s</td><td rowspan="4">72 °C for 7 min</td></tr>
<tr><td colspan="3">Touch town -1 °C/cycle, 10 cycles</td></tr>
<tr><td>95°C for 30 s</td><td>50 °C for 30 s</td><td>72 °C for 45 s</td></tr>
<tr><td colspan="3">25 cycles</td></tr>
</table>

Table S5. Description of the 22 plastid DNA haplotypes identified in *E. aphyllum*. Position of mutations in the base pairs (**†**) of the aligned *rps4*, *accd-trnE*(UUC) and *rps12-trnL*(GCA) matrix respectively. Condition found in the majority of samples is marked as "0". Values 4-8 in a position 206 of *rps12-trnL* alignment constitute number of repeats (n) of microsatellite motif $(TA)_n$.

**locus**	***rps4***	***accd-trnE*(UUC)**											***rps12-trnL*(GCA)**																	
**Haplotype**	**21†**	**138**	**152**	**155**	**319**	**341**	**343**	**350**	**353**	**380**	**462**	**473**	**75**	**87**	**104**	**122**	**192**	**202**	**206**	**247**	**297**	**303**	**314**	**426**	**445**	**483**	**491**	**494**	**504**	**537**
**H-1**	1	0	1	0	0	0	0	1	0	0	1	0	0	0	0	1	0	0	7	0	0	0	0	1	0	0	0	0	0	0
**H-2**	0	0	1	0	0	0	0	1	0	0	1	0	0	0	1	1	0	0	7	0	0	0	1	1	0	0	0	0	0	0
**H-3**	0	0	1	0	0	0	0	1	0	0	1	0	0	0	0	1	0	0	7	0	0	0	1	1	0	0	0	0	0	0
**H-4**	0	0	1	0	1	0	0	1	0	0	1	0	0	0	0	1	0	0	7	0	0	0	1	1	0	0	0	0	0	0
**H-5**	0	0	0	0	0	0	0	1	0	0	1	0	0	0	0	0	0	0	6	0	0	0	0	0	0	0	0	0	0	0
**H-6**	0	1	0	0	0	0	0	0	0	0	1	0	0	0	0	0	1	0	6	0	0	0	0	0	0	0	0	0	0	0
**H-7**	0	0	0	0	0	0	0	0	0	0	0	0	0	0	0	0	0	0	7	0	0	0	0	0	0	0	0	0	0	0
**H-8**	0	0	0	0	0	0	0	0	0	0	0	0	0	0	0	0	0	0	7	0	0	0	0	0	0	1	0	0	0	1
**H-9**	0	0	0	0	0	0	0	0	0	0	0	0	0	0	0	0	0	0	8	0	0	0	0	0	0	1	0	0	0	0
**H-10**	0	0	0	1	0	1	1	0	0	0	0	0	0	0	0	0	0	0	7	0	0	0	0	0	1	1	0	0	0	0
**H-11**	0	0	0	1	0	0	0	0	0	0	0	0	1	0	0	0	0	0	7	0	0	0	0	0	0	1	0	0	0	0
**H-12**	0	0	0	1	0	0	0	0	0	0	0	1	1	0	0	0	0	1	4	0	0	0	0	0	0	0	0	0	0	0
**H-13**	0	0	0	1	0	0	0	0	0	0	0	1	1	1	0	0	0	0	5	0	0	0	0	0	0	0	0	0	0	0
**H-14**	0	0	0	1	0	0	0	0	0	0	0	0	1	1	0	0	0	0	6	0	0	1	0	0	0	0	0	0	0	0
**H-15**	0	0	0	1	0	0	0	0	0	0	0	0	1	0	0	0	0	0	6	0	0	1	0	0	0	0	0	0	0	0
**H-16**	0	0	0	2	0	0	0	0	0	0	0	0	1	0	0	0	0	0	6	0	0	0	0	0	0	0	0	0	0	0
**H-17**	0	0	0	1	0	0	0	0	0	0	0	0	1	0	0	0	0	0	6	0	0	0	0	0	0	0	0	0	1	0
**H-18**	0	0	0	1	0	0	0	0	0	0	0	0	1	0	0	0	0	0	6	0	0	0	0	0	0	0	0	0	0	0
**H-19**	0	0	0	0	0	0	0	0	1	1	0	0	0	0	0	0	0	0	5	1	0	0	0	0	0	0	0	0	0	0
**H-20**	0	0	0	0	0	0	0	0	1	1	0	0	0	0	0	0	0	0	5	0	0	0	0	0	0	0	0	1	0	0
**H-21**	0	0	0	0	0	0	0	0	1	1	0	0	0	0	0	0	0	0	5	0	1	0	0	0	0	0	0	1	0	0
**H-22**	0	0	0	0	0	0	0	0	1	1	0	0	0	0	0	0	0	0	5	0	0	0	0	0	0	0	1	1	0	0

Table S6. Pairwise F_{ST} for nine populations of *E. aphyllum* based on nuclear SSR (lower diagonal) and plastid DNA (upper diagonal). Bolded values are statistically significant with P < 0.05

	TVF	MOF	BNF	VUS	OPP	TMP	JEE	LDR	ZDR
TVF		0.205	0.250	0.143	**0.306**	0.250	0.000	**1.000**	**0.344**
MOF	**0.121**		0.006	-0.002	0.126	0.076	0.080	**0.558**	**0.092**
BNF	**0.168**	0.052		-0.051	0.043	0.020	0.111	**0.500**	0.000
VUS	**0.290**	**0.106**	0.055		0.074	0.002	0.023	**0.456**	0.032
OPP	**0.132**	**0.086**	0.064	**0.095**		0.004	0.233	0.282	**0.097**
TMP	**0.166**	**0.109**	0.079	**0.113**	**0.100**		0.157	0.184	0.073
JEE	**0.288**	**0.169**	0.026	**0.089**	**0.123**	**0.121**		1.000	**0.256**
LDR	**0.184**	**0.118**	0.102	**0.119**	**0.106**	0.042	0.066		**0.397**
ZDR	**0.217**	**0.077**	0.006	0.055	**0.099**	**0.058**	**0.090**	**0.093**	

Table S7. Pairwise F_{ST} on a regional level *E. aphyllum* diversity based on nuclear SSR (lower diagonal) and plastid DNA (upper diagonal). Bolded values are statistically significant with P < 0.05.

	Pyrenees	Masssif Central	Alps	Carpathians	North Europe	East
Pyrenees		0.179	**0.189**	**0.275**	0.238	**0.438**
Masssif Central	0.033		-0.004	0.064	0.057	**0.128**
Alps	**0.110**	**0.060**		0.024	0.076	0.048
Carpathians	**0.058**	**0.050**	**0.035**		**0.075**	0.062
North Europe	**0.070**	**0.049**	0.024	0.013		**0.188**
Asia	**0.151**	**0.090**	0.027	**0.056**	0.044	

Table S8. Identification of symbiotic fungal species of *E. aphyllum* found in present study. For the species of *Inocybe* their affiliation to the alignment groups (AG) according to Ryberg *et al*. (2009) is given. WEP- Wejherowo, TMP- Tatra Mountains, KRP - Kozi Rynek, OPP - Ojcowski National Park.

allignment group	OTU	Population	Sample name	GenBank accesion No	The most similar sequence in UNITE (UDB) or GenBank : species. (accesion No)	E value	Maximum identity (%)
AG-2	OTU3	WEP*	WEP_Ea202	KX867466	*Inocybe glabrescens* Velen. (HQ604513)	0.0	98
	OTU8	WEP	WEP_Ea482<br>WEP_Ea93	KX867483<br>KX867484	*Inocybe nitidiuscula* (Britzelm.) Lapl. (AM882913)	0.0	99
		KRP	KRP_Ea1	OL461961			
	OTU9	WEP	WEP_Ea507	KX867485	*Inocybe flocculosa* (Berk.) Sacc.(AM882992)	0.0	99
	OTU15	TMP	TMP_Ea17-2	OL461963	*Inocybe sindonia* (Fr.) P. Karst. (HQ604384)	0.0	99
AG-3	OTU14	TMP	TMP_Ea17-4<br>TMP_Ea17-5	OL461964<br>OL461965	*Inocybe cervicolor* (Pers.) Quél. (UDB032065)	0.0	98
AG-5	OTU1	WEP	WEP_Ea125	KX867464	*Inocybe lilacina* (Peck) Kauffman (JF908236)	0.0	100
	OTU5	WEP	WEP_Ea256	KX867468	*Inocybe posterula* (Britzelm.) Sacc. (JF908223)	0.0	99

AG-7	OTU13	WEP	WEP_Ea429<br>WEP_Ea460	KX867502<br>KX867503	*Inocybe subbrunnea* Kühner (KJ399945)	0.0	99
		TMP	TMP_Ea17-1	OL461962			
	OTU11	WEP	WEP_Ea234	KX867492	*Inocybe cf castanea* Velenovsky (HQ604374)	0.0	99
AG-8	OTU7	WEP	WEP_Ea34	KX867482	*Inocybe griseolilacina* J.E. Lange (AM882728)	0.0	99
	OTU4	WEP	WEP_Ea218	KX867467	*Inocybe subnudipes* Kühner (UDB015353)	0.0	99
AG-13	OTU2	WEP	WEP_Ea194-P2	KX867465	*Inocybe cf. mixtilis* (Britzelm.) Sacc. (AM882836)	0.0	99
	OTU10	WEP	WEP_Ea115, WEP_Ea154, WEP_Ea162, WEP_Ea226, WEP_Ea233, WEP_Ea346, WEP_Ea192, WEP_Ea406	KX867486 to KX867494	*Incyte mixtilis* (Britzelm.) Sacc (HQ604488)	0.0	100
AG-16	OTU6	WEP	WEP_Ea277, WEP_Ea279, WEP_Ea214, WEP_Ea264, WEP_Ea300, WEP_Ea342, WEP_Ea453, WEP_Ea457, WEP_Ea87, WEP_EaAlb, WEP_Ea194-P1, WEP_Ea501	KX867469 to KX867481	*Inocybe terrigena* Fr. Kühner (AM882864)	0.0	99
		TMP	TMP_Ea14-2	KX940905			

	OTU12	WEP	WEP_Ea441, WEP_Ea182, WEP_Ea119, WEP_Ea171, WEP_Ea380, WEP_Ea287, WEP_Ea297, WEP_EaRos	KX867495 to KX867494	*Inocybe leucoblema* Kühner (HM209789)	0.0	99
	OTU16	OPP	OPP_Ea17-2, OPP_Ea17-3, OPP_Ea17-4, OPP_Ea17-5,	OL461967 to OL461970	*Hebeloma sinapizans* (Paulet) Gillet (HF678206)	0.0	99
	OTU17	TMP	TMP_Ea17-3	OL461966	*Hebeloma incarnatulum* A. H. Sm. (AF430291)	0.0	99
	OUT 18	OPP	OPP_Ea17-4T	OL461971	*Tomentellopsis sp* (AJ893355)	0.0	94

*40 specimens of *E. aphyllum* from WEP represent the same multilocus genotype.
